# Supplementary figures and images for: A universal pocket in fatty acyl-AMP ligases ensures redirection of fatty acid pool away from coenzyme A-based activation
Source: eLife. 2021 Sep 7;10:e70067. doi: 10.7554/eLife.70067 (PMC8460268; doi:10.7554/eLife.70067)

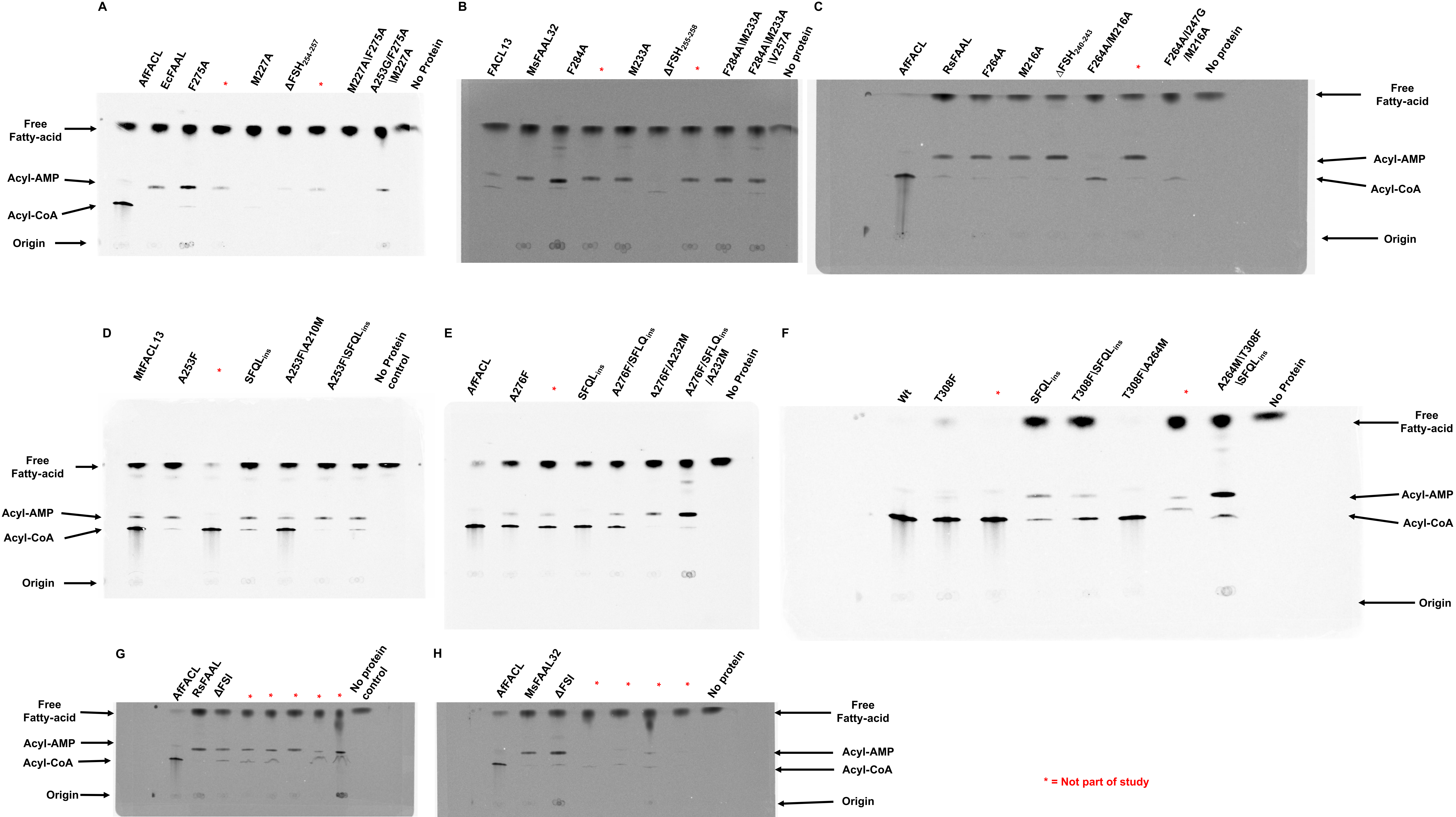

Supplement: Figure 2—source data 1. — It was also used to compare the gain of function obtained from deletion of the previously annotated FAAL-specific insertion (ΔFSI) and FAAL-specific helix (ΔFSH). Wild-type FAAL, wild-type FACL, and reaction lacking any protein were used as controls. All the TLCs were marked at the origin, where the reaction mix containing 1-14C fatty acids was spotted. The products acyl-CoA band and the acyl-AMP band along with the free fatty acid band are visualized owing to the radio-labeled fatty acid. (A) A representative image of TLC showing the canonical pocket mutations leading to the gain of function in EcFAAL. (B) A representative image of TLC showing the canonical pocket mutations leading to the gain of function in MsFAAL32. (C) A representative image of TLC showing the canonical pocket mutations leading to the gain of function in RsFAAL. (D) A representative image of TLC showing the canonical pocket mutations leading to the loss of function in MtFACL13. (E) A representative image of TLC showing the canonical pocket mutations leading to the loss of function in AfFACL. (F) A representative image of TLC showing the canonical pocket mutations leading to the loss of function in EcFACL. (G) A representative image of TLC showing the gain of function by ΔFSI mutation in RsFAAL. (H) A representative image of TLC showing the gain of function by ΔFSI mutation in MsFAAL32. Several mutations were generated in this study, which had multiple issues including protein stability, poor or complete loss of biochemical activity, etc., hence were not analyzed further and such mutations are marked by a red asterisk as ‘not part of the study.’ These original uncropped images of radio-TLC are source data for Figure 2. [file elife-70067-fig2-data1.pdf]

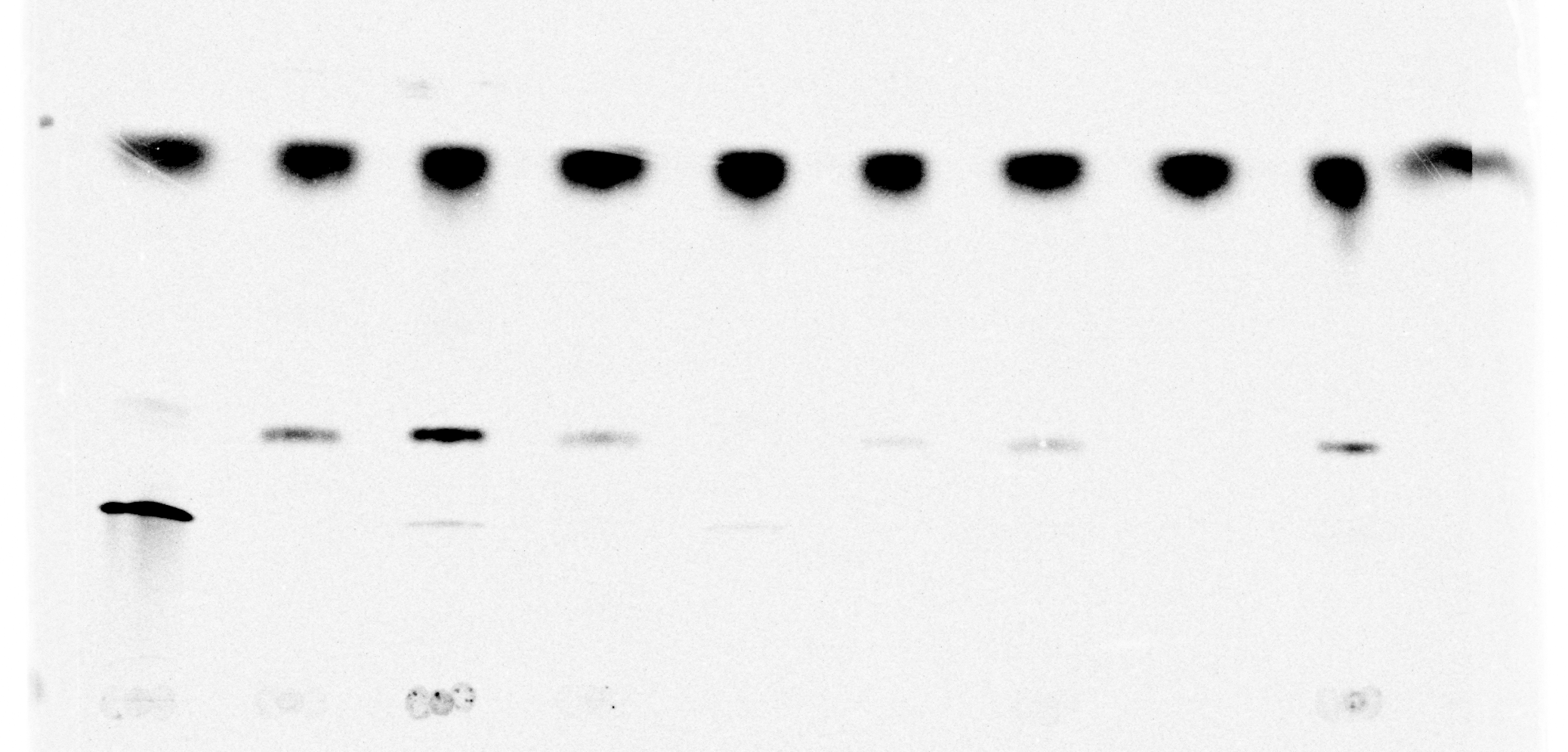

Supplement: Figure 2—source data 5. [file elife-70067-fig2-data5.zip › Figure 2 source data1-a.png]

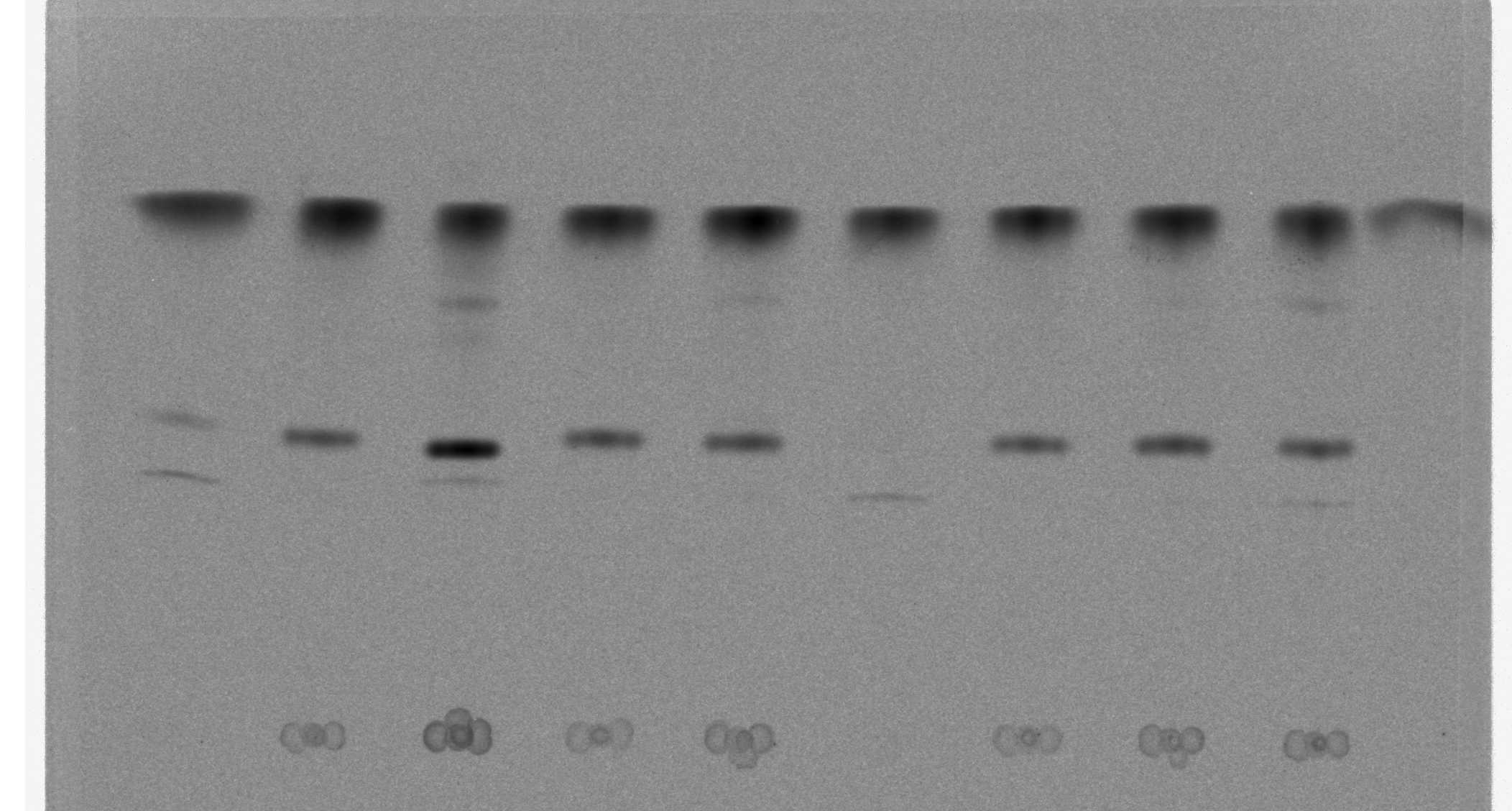

Supplement: Figure 2—source data 5. [file elife-70067-fig2-data5.zip › Figure 2 source data1-b.png]

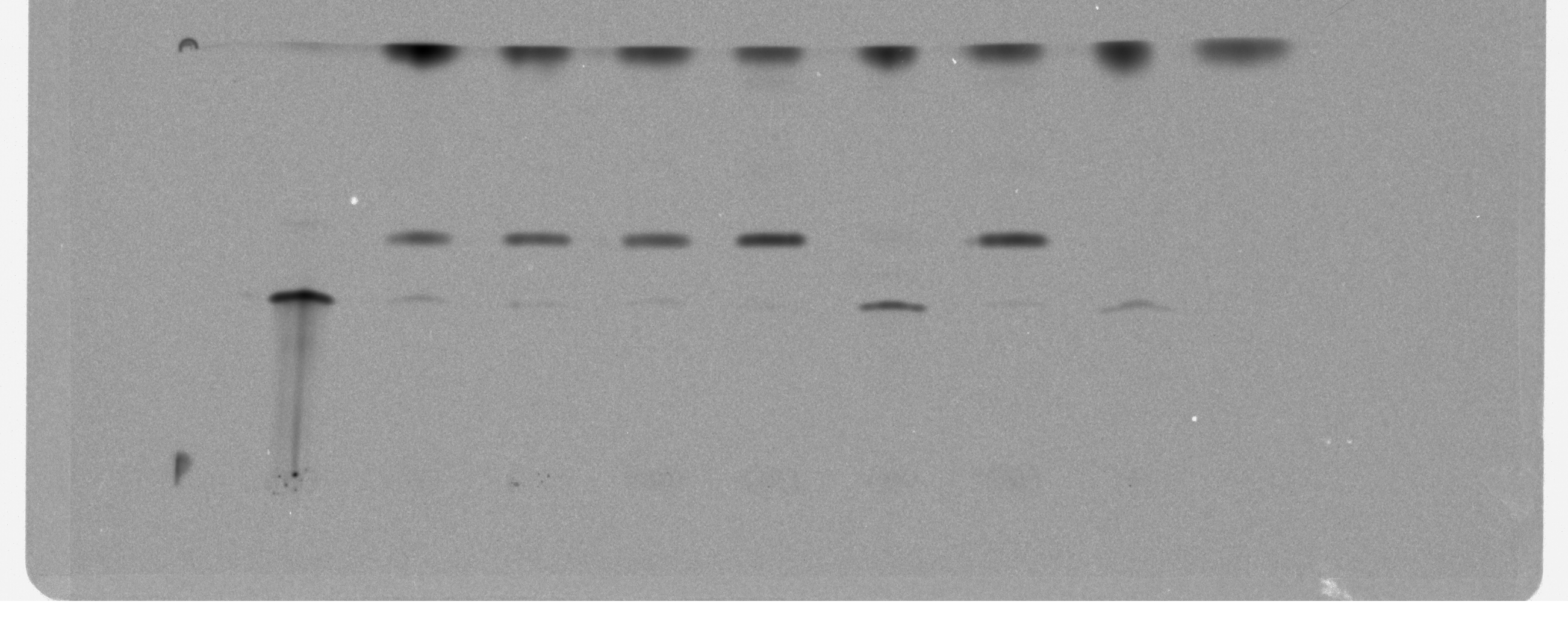

Supplement: Figure 2—source data 5. [file elife-70067-fig2-data5.zip › Figure 2 source data1-c.png]

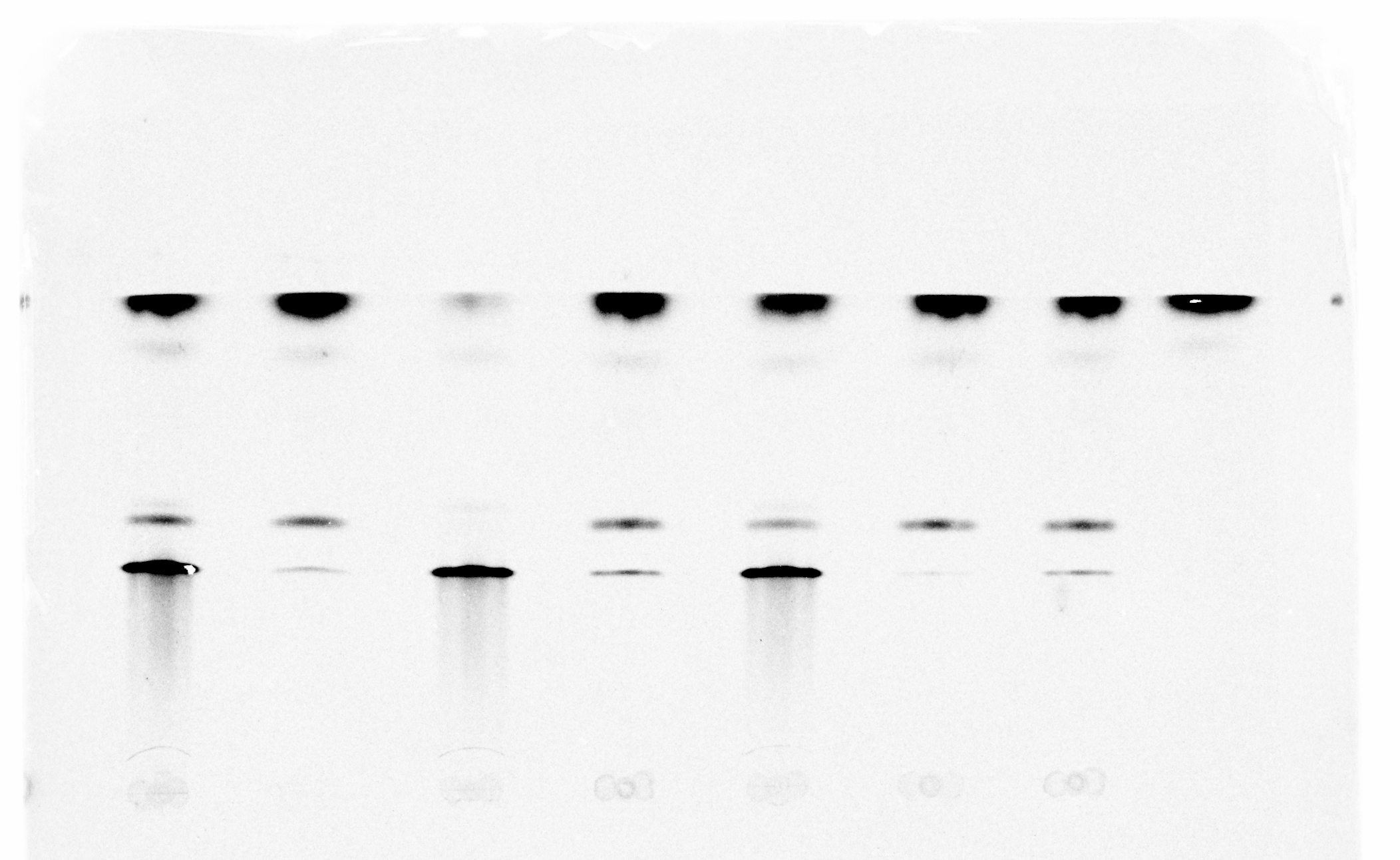

Supplement: Figure 2—source data 5. [file elife-70067-fig2-data5.zip › Figure 2 source data1-d.png]

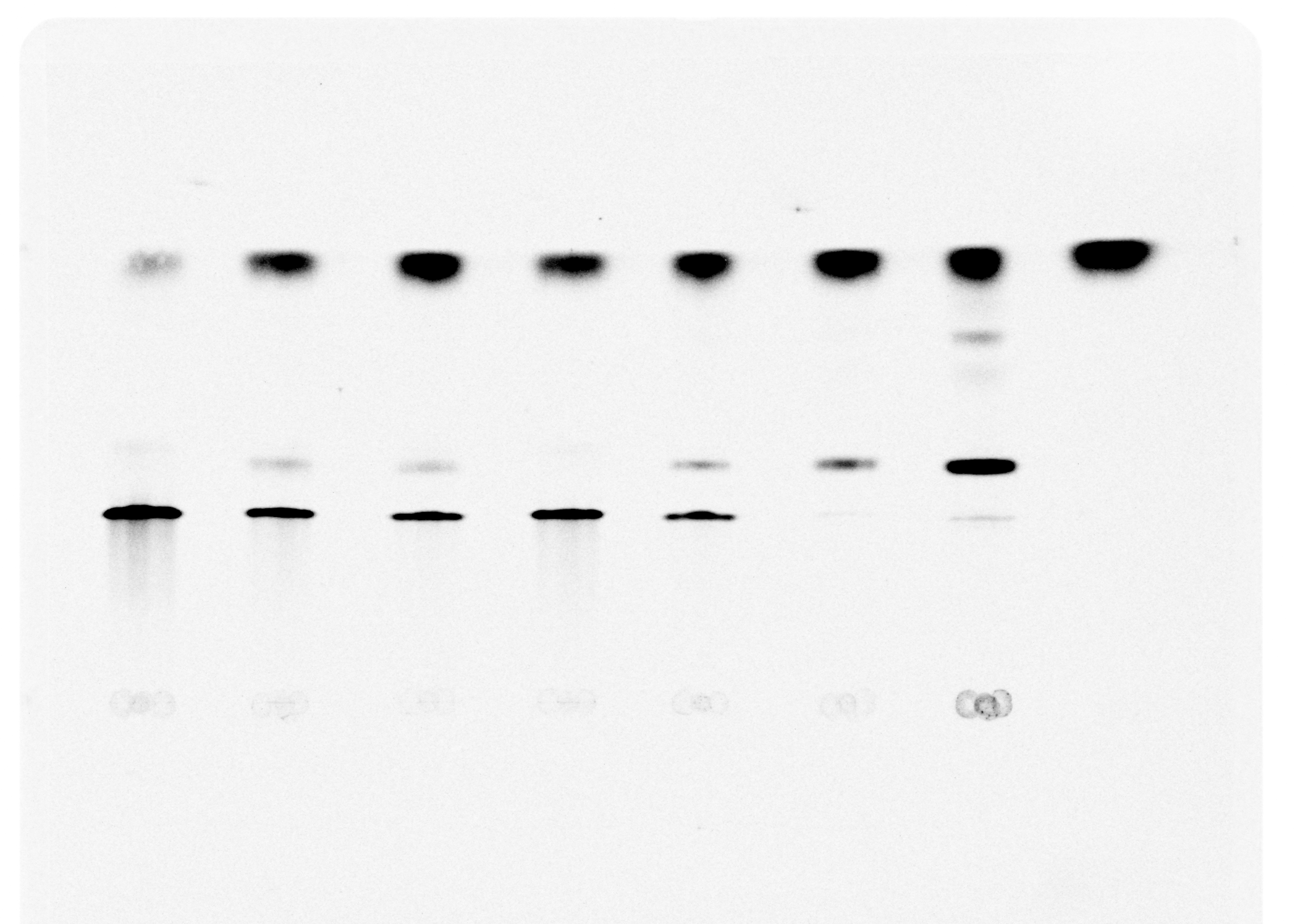

Supplement: Figure 2—source data 5. [file elife-70067-fig2-data5.zip › Figure 2 source data1-e.png]

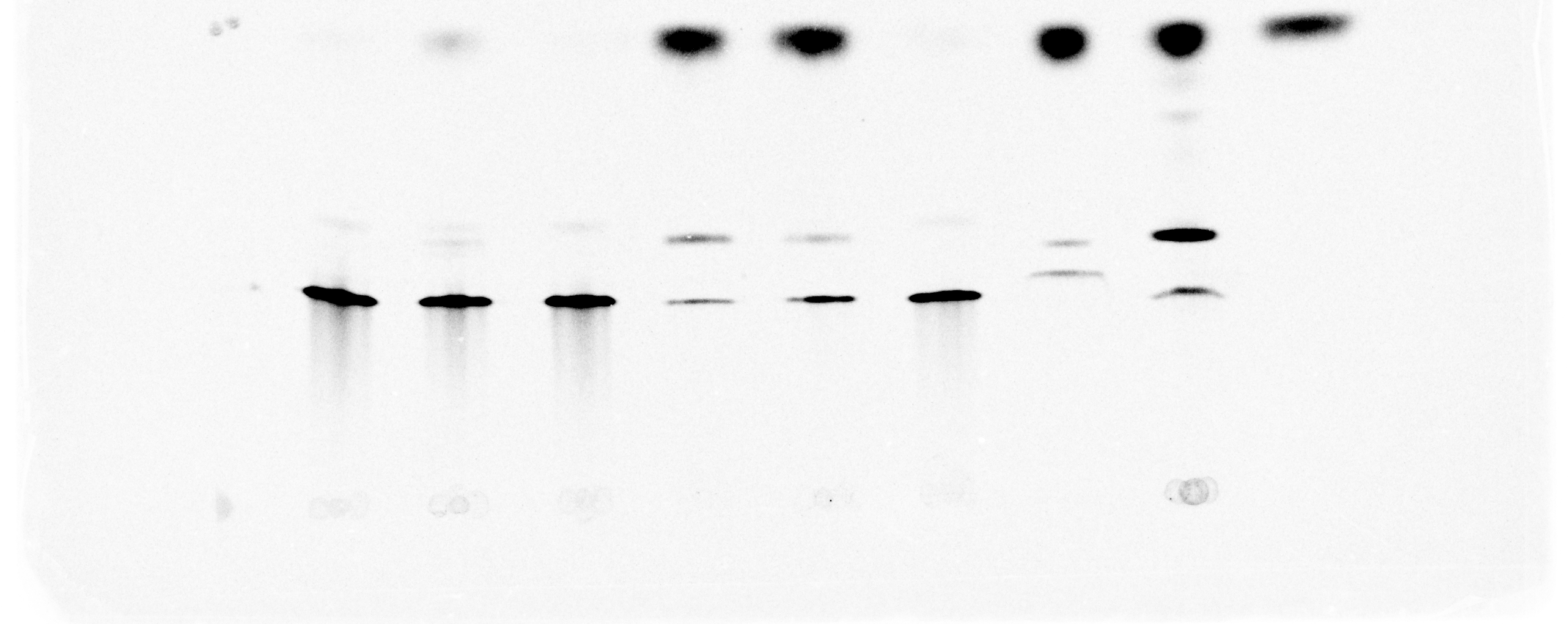

Supplement: Figure 2—source data 5. [file elife-70067-fig2-data5.zip › Figure 2 source data1-f.png]

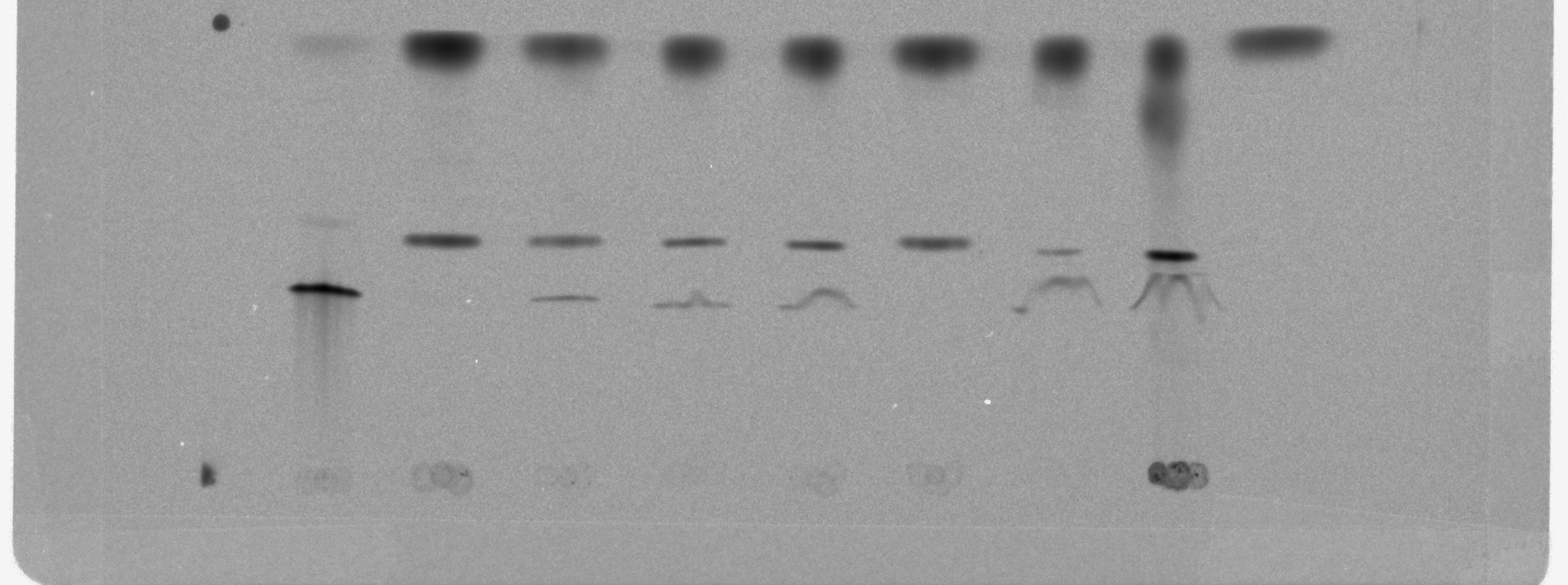

Supplement: Figure 2—source data 5. [file elife-70067-fig2-data5.zip › Figure 2 source data1-g.png]

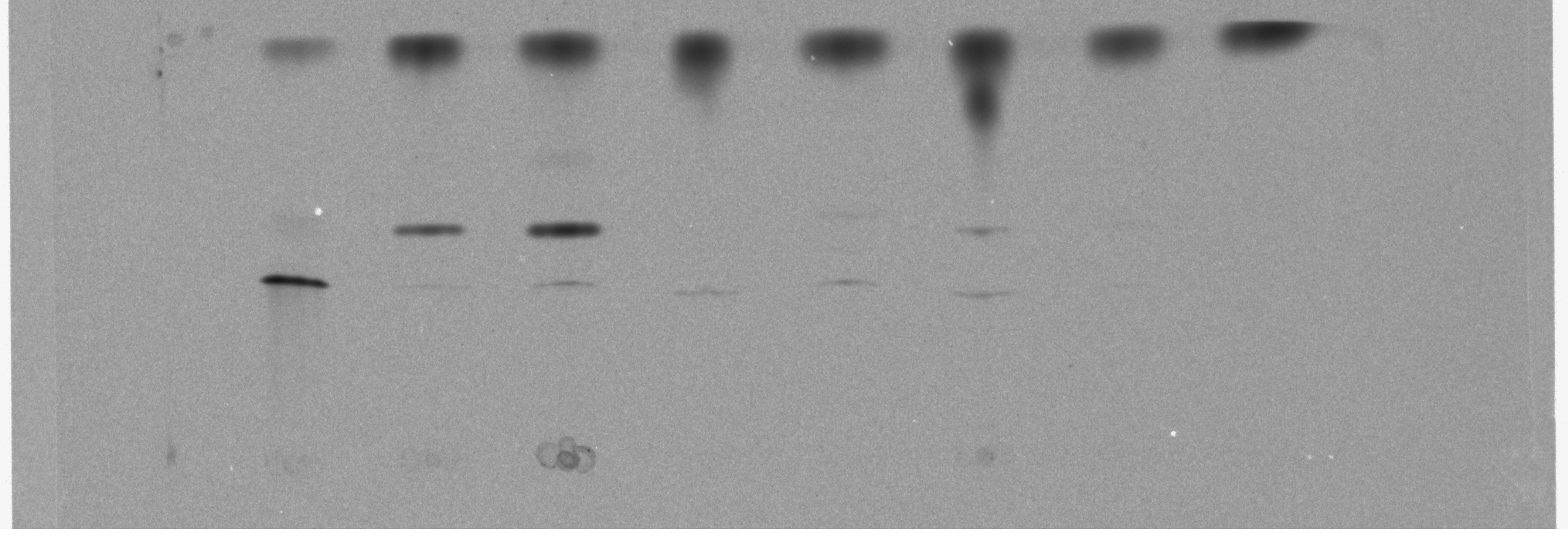

Supplement: Figure 2—source data 5. [file elife-70067-fig2-data5.zip › Figure 2 source data1-h.png]

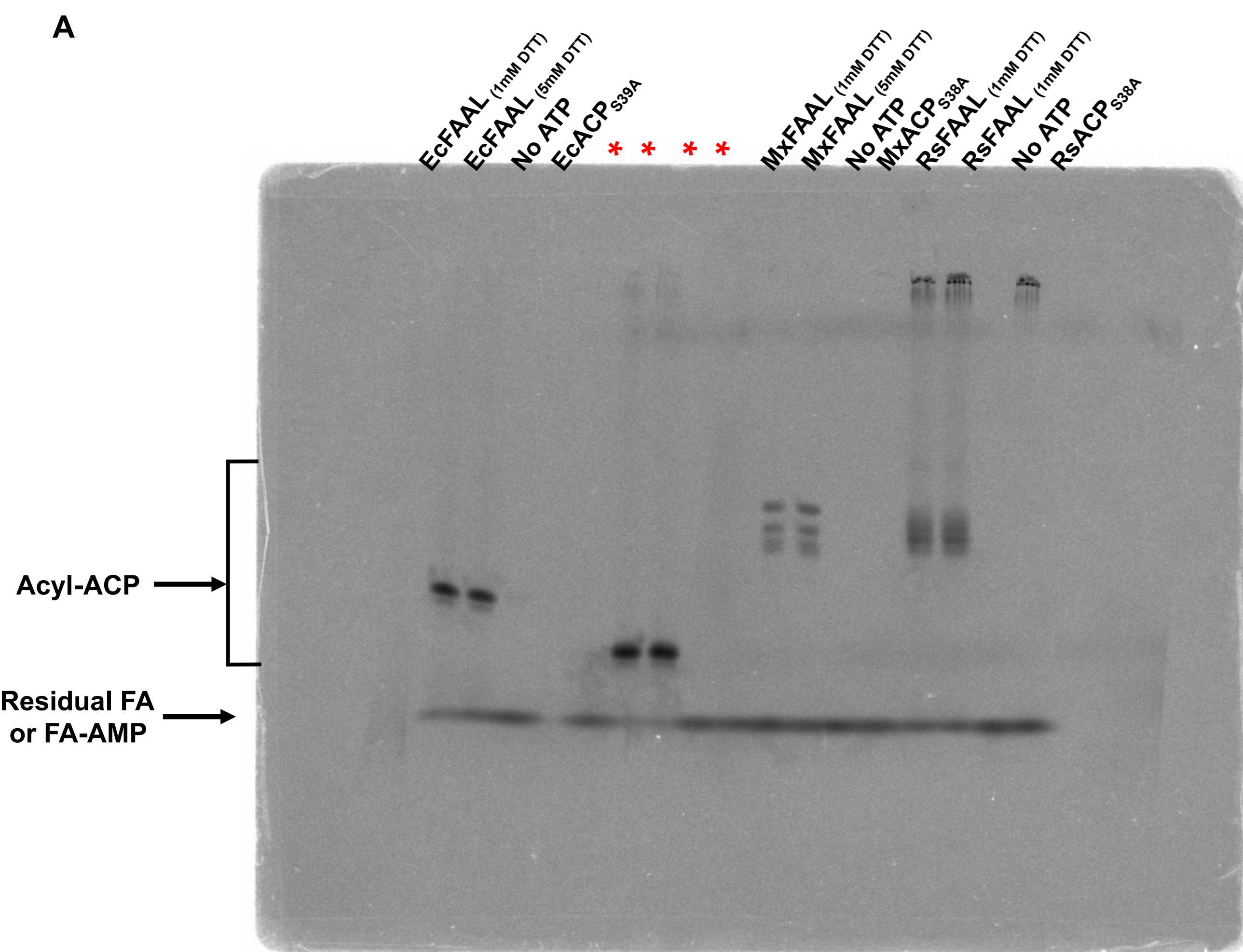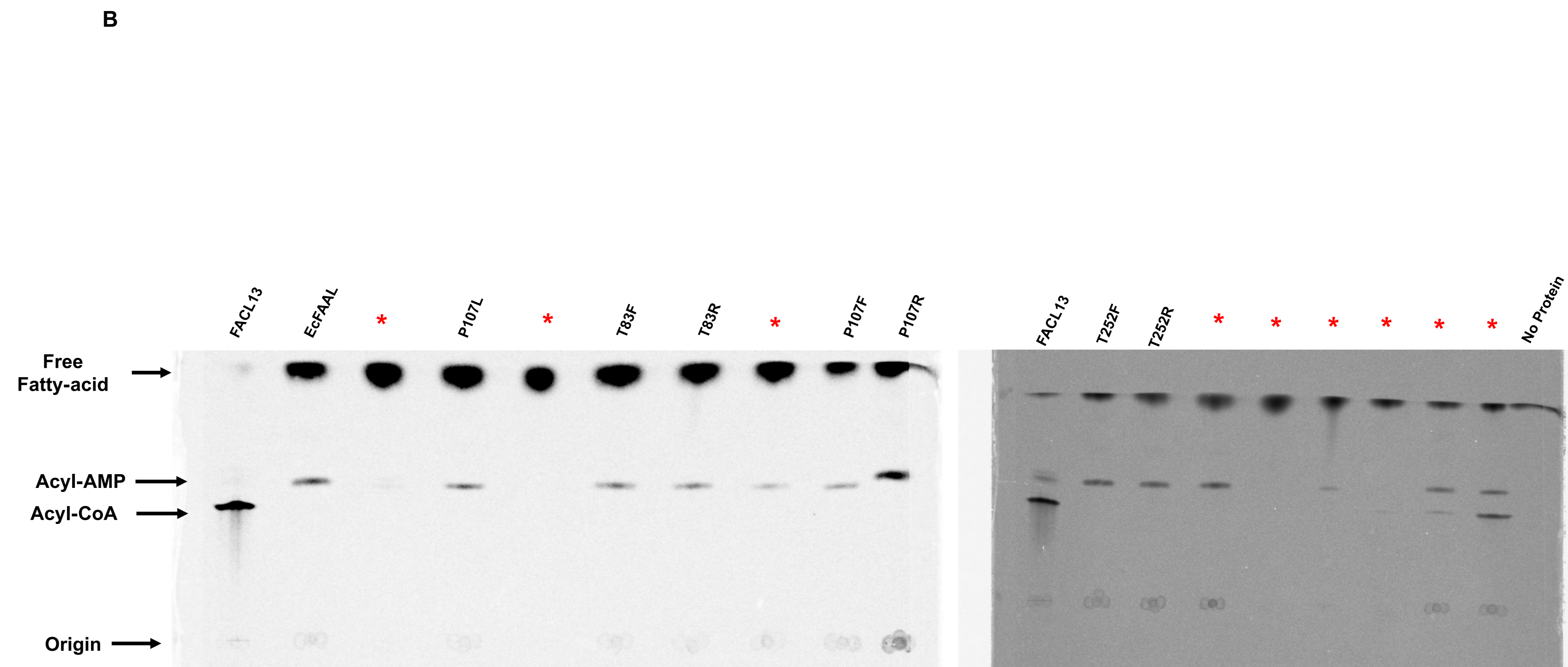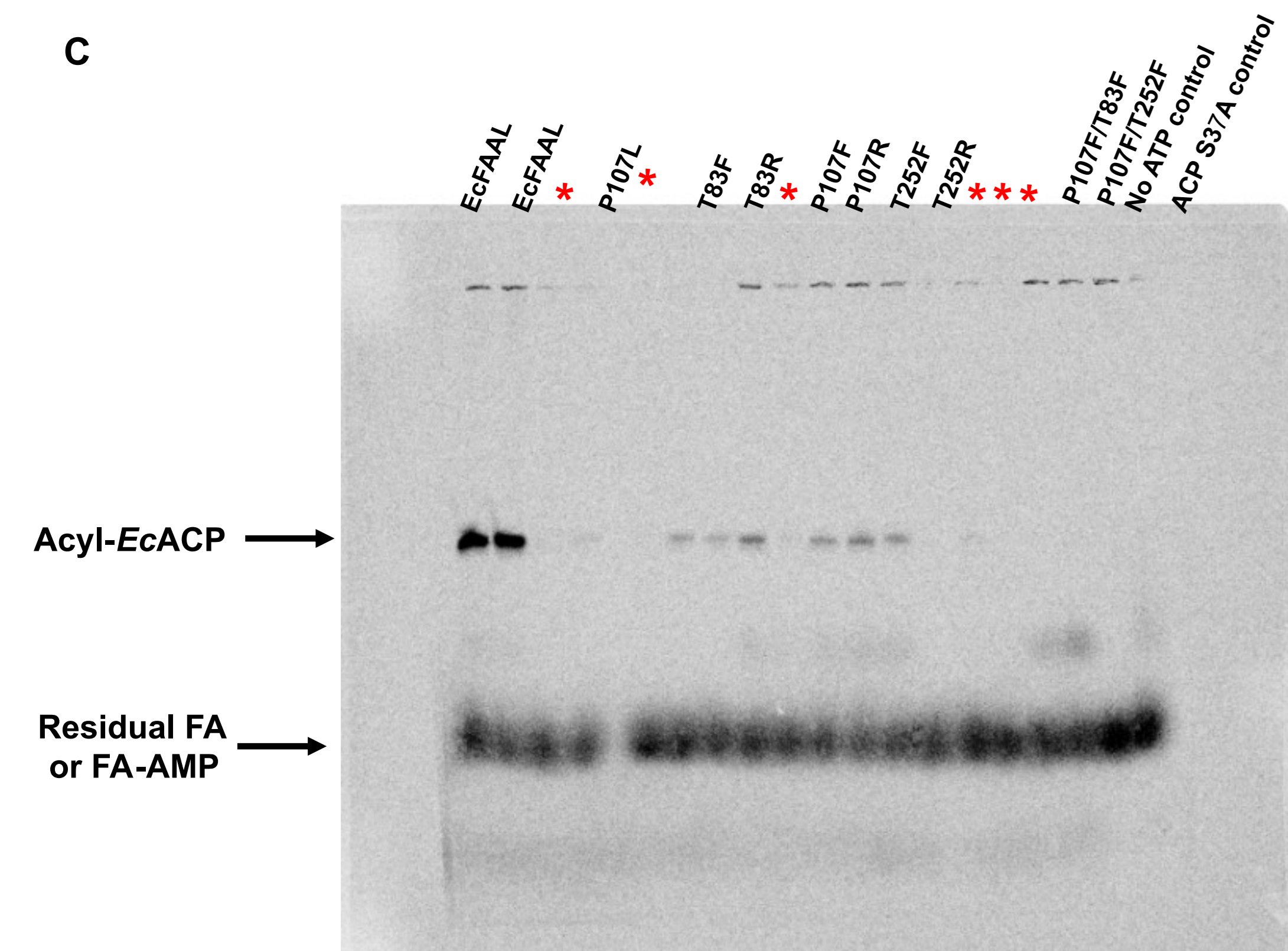

\* Not part of this study

Supplement: Figure 4—source data 1. — These were also used to assess the impact of mutations in the alternative pocket of EcFAAL to form acyl-EcACP. Wild-type FAAL, reaction lacking ATP, and reaction containing a mutant ACP, lacking 4'-phosphopantetheine arm, were used as controls. All the radio-CS-PAGE were marked at the origin, where the reaction mix containing 1-14C fatty acids was loaded without boiling. The acyl-ACP(s), along with unreacted fatty acids or acyl-AMP(s), could only be visualized owing to the radiolabeled fatty acid. The unreacted fatty acids or acyl-AMP(s) appeared as a diffused band at the bottom of the radio-CS-PAGE. The radio-TLC images presented here were used to assess the ability of EcFAAL and its mutations in the alternate pocket to form acyl-AMP. Wild-type EcFAAL, wild-type MtFACL13, and reaction lacking any protein were used as controls. All the TLCs were marked at the origin, where the reaction mix containing 1-14C fatty acids was spotted. The products, acyl-CoA band and the acyl-AMP band, along with the free fatty acid band, were visualized owing to the radiolabeled fatty acid. (A) A representative image showing the optimization and validation of the modiﬁed radio-CS-PAGE in three pairs of FAAL-ACP systems, namely EcFAAL-EcACP, RsFAAL-RsACP, and MxFAAL-MxACP. (B) A representative TLC image showing that alternate pocket mutations of EcFAAL have minimal or no eﬀect on the acyl-AMP formation. (C) A representative image of radio-CS-PAGE showing that acyl-EcACP formation is severely attenuated by mutations in the alternate pocket of EcFAAL. Several mutations of the alternate pocket of EcFAAL were generated in this study, which had multiple issues including protein stability, poor or complete loss of biochemical activity, etc., hence were not analyzed further, and these mutations are marked by a red asterisk as ‘not part of the study.’ These original uncropped images of radio-CS-PAGE and radio-TLC are source data for Figure 4. [file elife-70067-fig4-data1.pdf]

|                    |   |   |   |   |   |   |
|--------------------|---|---|---|---|---|---|
| Wt <i>Ec</i> FAAL  | + | + | + | - | + | + |
| <i>holo-Ec</i> ACP | - | + | + | + | + | - |
| FA                 | + | + | + | - | - | - |
| ATP                | + | - | + | - | - | - |

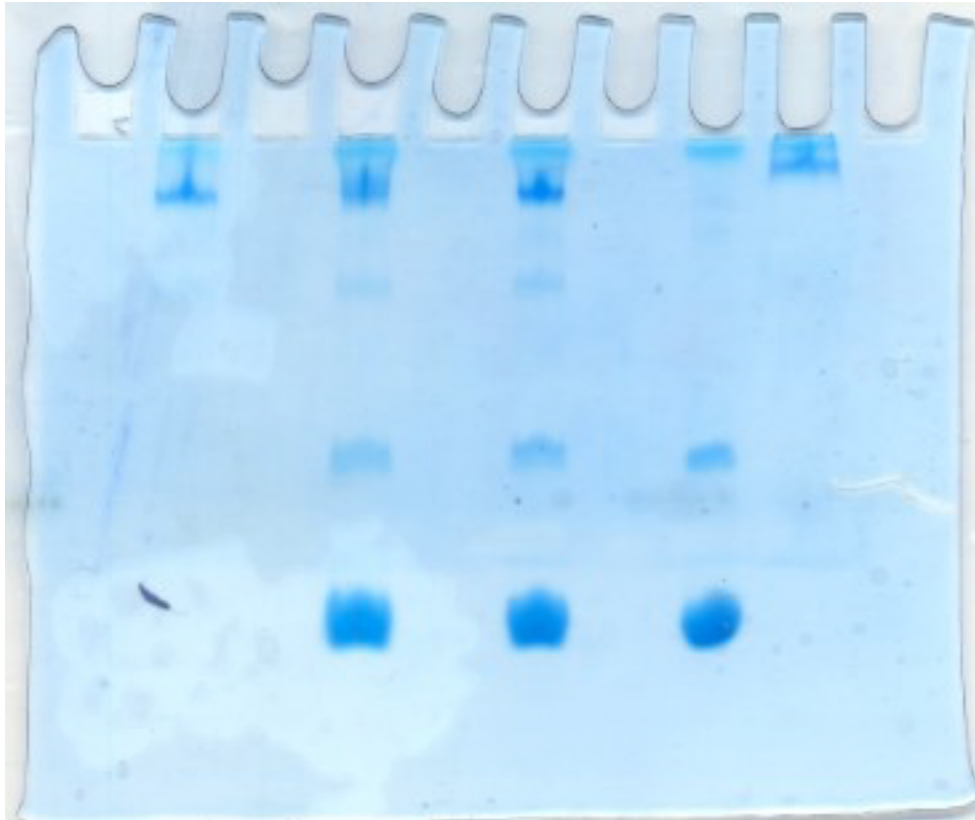

→ *holo*-ACP or *apo*-ACP  
or acyl-ACP

Supplement: Figure 4—figure supplement 1—source data 1. — The Coomassie-stained gel does not reveal the classic separation of holo-ACP, acyl-ACP, and apo-ACP. [file elife-70067-fig4-figsupp1-data1.pdf]

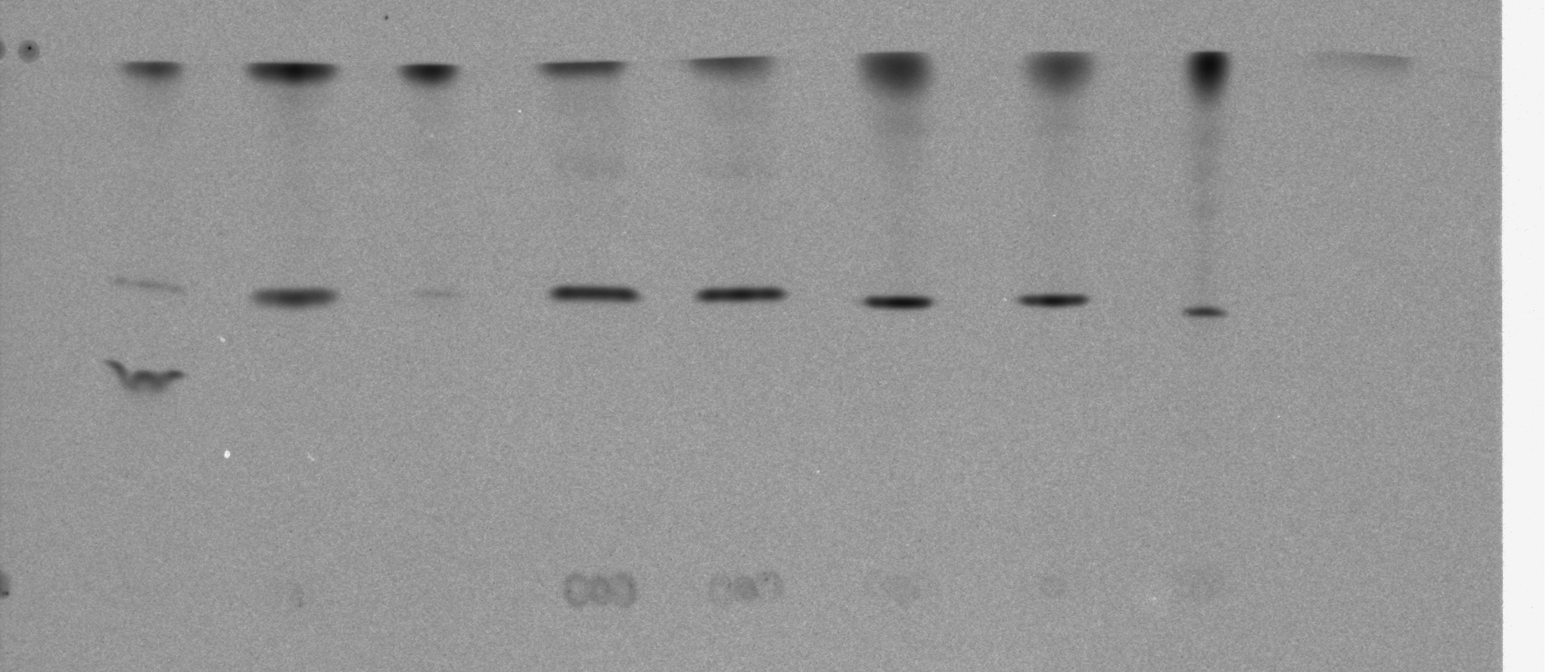

Supplement: Figure 4—figure supplement 2—source data 1. — All the TLCs are marked with the origin (where 20 μL reaction mix is spotted), the acyl-CoA band (closest to origin), the acyl-AMP band (closest to the solvent front), and the free fatty acid band (near the solvent front). All the modified radio-CS-PAGE are marked with the acyl-ACP band and a diffused band, which may be free fatty acids or the acyl-AMP formed in the reaction. (A) Representative TLC images showing that the acyl-AMP formation is minimally or not affected by mutations in the alternate pocket of MxFAAL. (B) A representative modified radio-CS-PAGE image showing the acyl-MxACP formation by alternate pocket mutants of MxFAAL. The multiple bands in the modified radio-CS-PAGE may represent either the degradation products of MxACP-GFP or the multiple unfolded forms of MxACP-GFP in the presence of urea in the gel. (C) Representative TLC images showing the acyl-AMP formation by alternate pocket mutants of RsFAAL. (D) A representative modified radio-CS-PAGE image showing the acyl-RsACP formation by alternate pocket mutants of RsFAAL. (E) Representative TLC images showing the acyl-AMP formation by alternate pocket mutants of MsFAAL32. (F) A representative radio-SDS-PAGE showing the acyl-MsPKS131-1042 formation by the alternate pocket mutants of MsFAAL32. Several mutations of the alternate pocket of different FAALs were generated in this study, which had multiple issues including protein stability, poor or complete loss of biochemical activity, etc., hence were not discussed further, and these mutations are marked by a red asterisk as ‘not part of the study.’. [file elife-70067-fig4-figsupp2-data1.zip › Figure 4—Figure_Supplement_2_source_data1-c1.png]

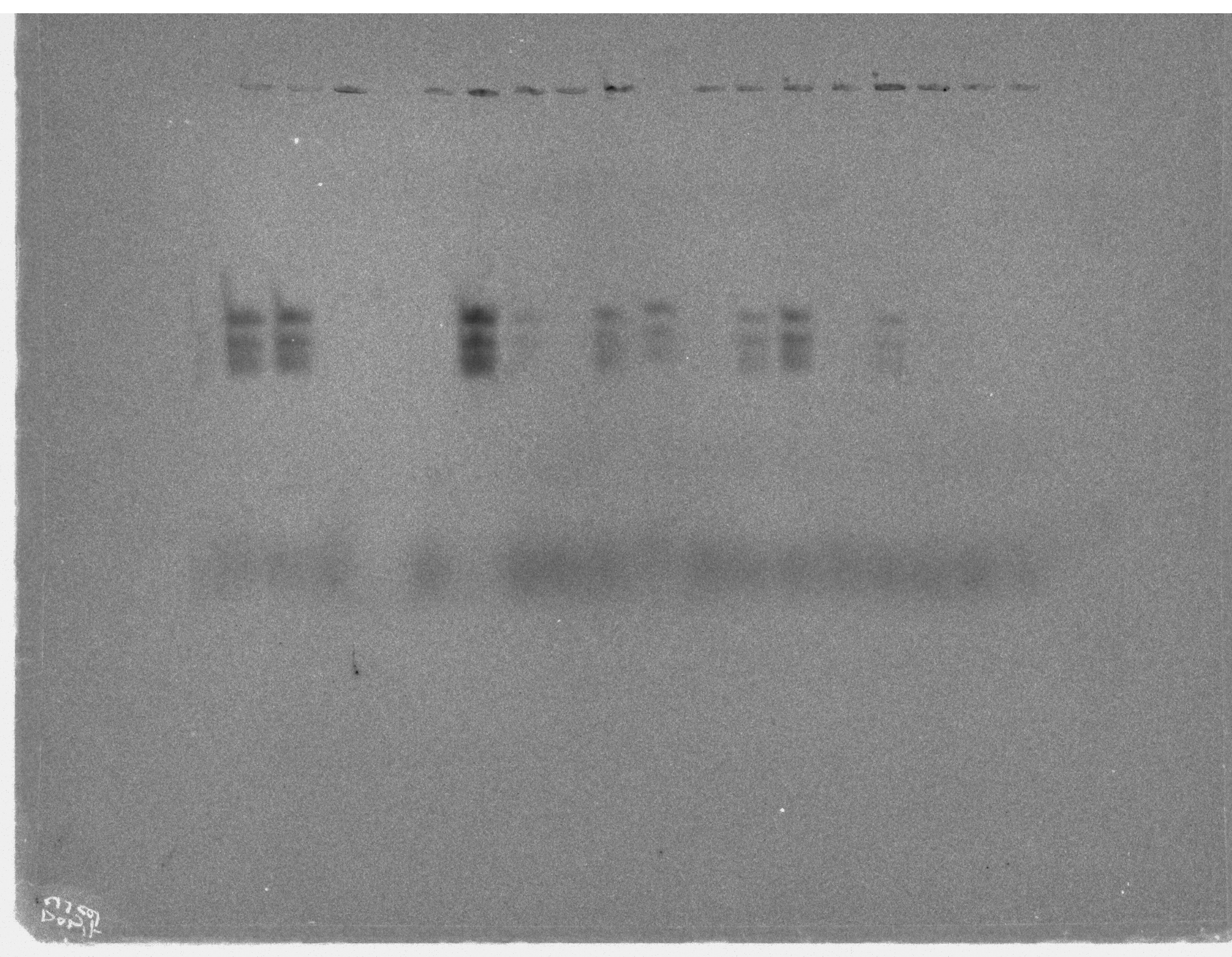

Supplement: Figure 4—figure supplement 2—source data 1. — All the TLCs are marked with the origin (where 20 μL reaction mix is spotted), the acyl-CoA band (closest to origin), the acyl-AMP band (closest to the solvent front), and the free fatty acid band (near the solvent front). All the modified radio-CS-PAGE are marked with the acyl-ACP band and a diffused band, which may be free fatty acids or the acyl-AMP formed in the reaction. (A) Representative TLC images showing that the acyl-AMP formation is minimally or not affected by mutations in the alternate pocket of MxFAAL. (B) A representative modified radio-CS-PAGE image showing the acyl-MxACP formation by alternate pocket mutants of MxFAAL. The multiple bands in the modified radio-CS-PAGE may represent either the degradation products of MxACP-GFP or the multiple unfolded forms of MxACP-GFP in the presence of urea in the gel. (C) Representative TLC images showing the acyl-AMP formation by alternate pocket mutants of RsFAAL. (D) A representative modified radio-CS-PAGE image showing the acyl-RsACP formation by alternate pocket mutants of RsFAAL. (E) Representative TLC images showing the acyl-AMP formation by alternate pocket mutants of MsFAAL32. (F) A representative radio-SDS-PAGE showing the acyl-MsPKS131-1042 formation by the alternate pocket mutants of MsFAAL32. Several mutations of the alternate pocket of different FAALs were generated in this study, which had multiple issues including protein stability, poor or complete loss of biochemical activity, etc., hence were not discussed further, and these mutations are marked by a red asterisk as ‘not part of the study.’. [file elife-70067-fig4-figsupp2-data1.zip › Figure 4—Figure_Supplement_2_source_data1-b.png]

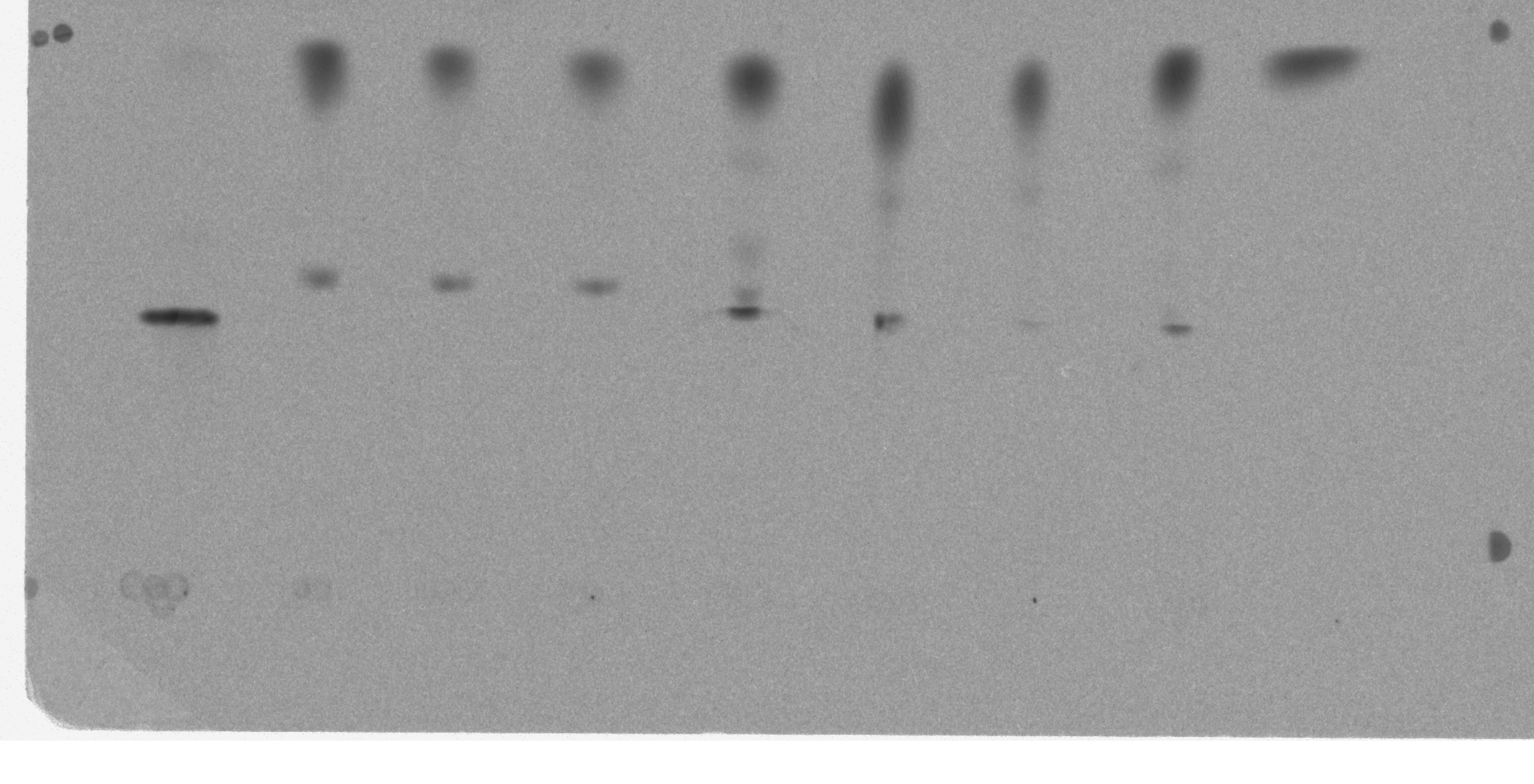

Supplement: Figure 4—figure supplement 2—source data 1. — All the TLCs are marked with the origin (where 20 μL reaction mix is spotted), the acyl-CoA band (closest to origin), the acyl-AMP band (closest to the solvent front), and the free fatty acid band (near the solvent front). All the modified radio-CS-PAGE are marked with the acyl-ACP band and a diffused band, which may be free fatty acids or the acyl-AMP formed in the reaction. (A) Representative TLC images showing that the acyl-AMP formation is minimally or not affected by mutations in the alternate pocket of MxFAAL. (B) A representative modified radio-CS-PAGE image showing the acyl-MxACP formation by alternate pocket mutants of MxFAAL. The multiple bands in the modified radio-CS-PAGE may represent either the degradation products of MxACP-GFP or the multiple unfolded forms of MxACP-GFP in the presence of urea in the gel. (C) Representative TLC images showing the acyl-AMP formation by alternate pocket mutants of RsFAAL. (D) A representative modified radio-CS-PAGE image showing the acyl-RsACP formation by alternate pocket mutants of RsFAAL. (E) Representative TLC images showing the acyl-AMP formation by alternate pocket mutants of MsFAAL32. (F) A representative radio-SDS-PAGE showing the acyl-MsPKS131-1042 formation by the alternate pocket mutants of MsFAAL32. Several mutations of the alternate pocket of different FAALs were generated in this study, which had multiple issues including protein stability, poor or complete loss of biochemical activity, etc., hence were not discussed further, and these mutations are marked by a red asterisk as ‘not part of the study.’. [file elife-70067-fig4-figsupp2-data1.zip › Figure 4—Figure_Supplement_2_source_data1-a2.png]

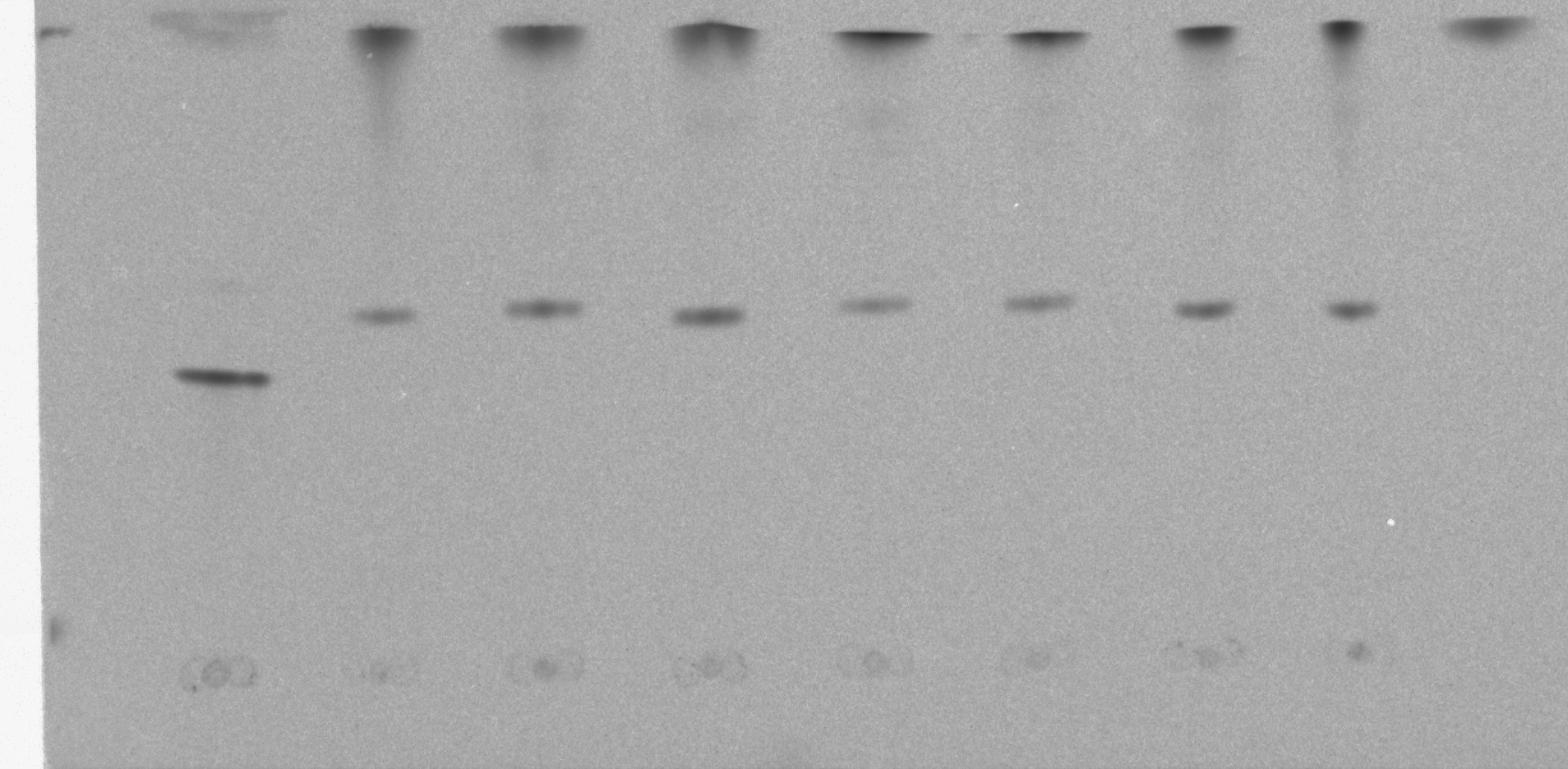

Supplement: Figure 4—figure supplement 2—source data 1. — All the TLCs are marked with the origin (where 20 μL reaction mix is spotted), the acyl-CoA band (closest to origin), the acyl-AMP band (closest to the solvent front), and the free fatty acid band (near the solvent front). All the modified radio-CS-PAGE are marked with the acyl-ACP band and a diffused band, which may be free fatty acids or the acyl-AMP formed in the reaction. (A) Representative TLC images showing that the acyl-AMP formation is minimally or not affected by mutations in the alternate pocket of MxFAAL. (B) A representative modified radio-CS-PAGE image showing the acyl-MxACP formation by alternate pocket mutants of MxFAAL. The multiple bands in the modified radio-CS-PAGE may represent either the degradation products of MxACP-GFP or the multiple unfolded forms of MxACP-GFP in the presence of urea in the gel. (C) Representative TLC images showing the acyl-AMP formation by alternate pocket mutants of RsFAAL. (D) A representative modified radio-CS-PAGE image showing the acyl-RsACP formation by alternate pocket mutants of RsFAAL. (E) Representative TLC images showing the acyl-AMP formation by alternate pocket mutants of MsFAAL32. (F) A representative radio-SDS-PAGE showing the acyl-MsPKS131-1042 formation by the alternate pocket mutants of MsFAAL32. Several mutations of the alternate pocket of different FAALs were generated in this study, which had multiple issues including protein stability, poor or complete loss of biochemical activity, etc., hence were not discussed further, and these mutations are marked by a red asterisk as ‘not part of the study.’. [file elife-70067-fig4-figsupp2-data1.zip › Figure 4—Figure_Supplement_2_source_data1-a1.png]

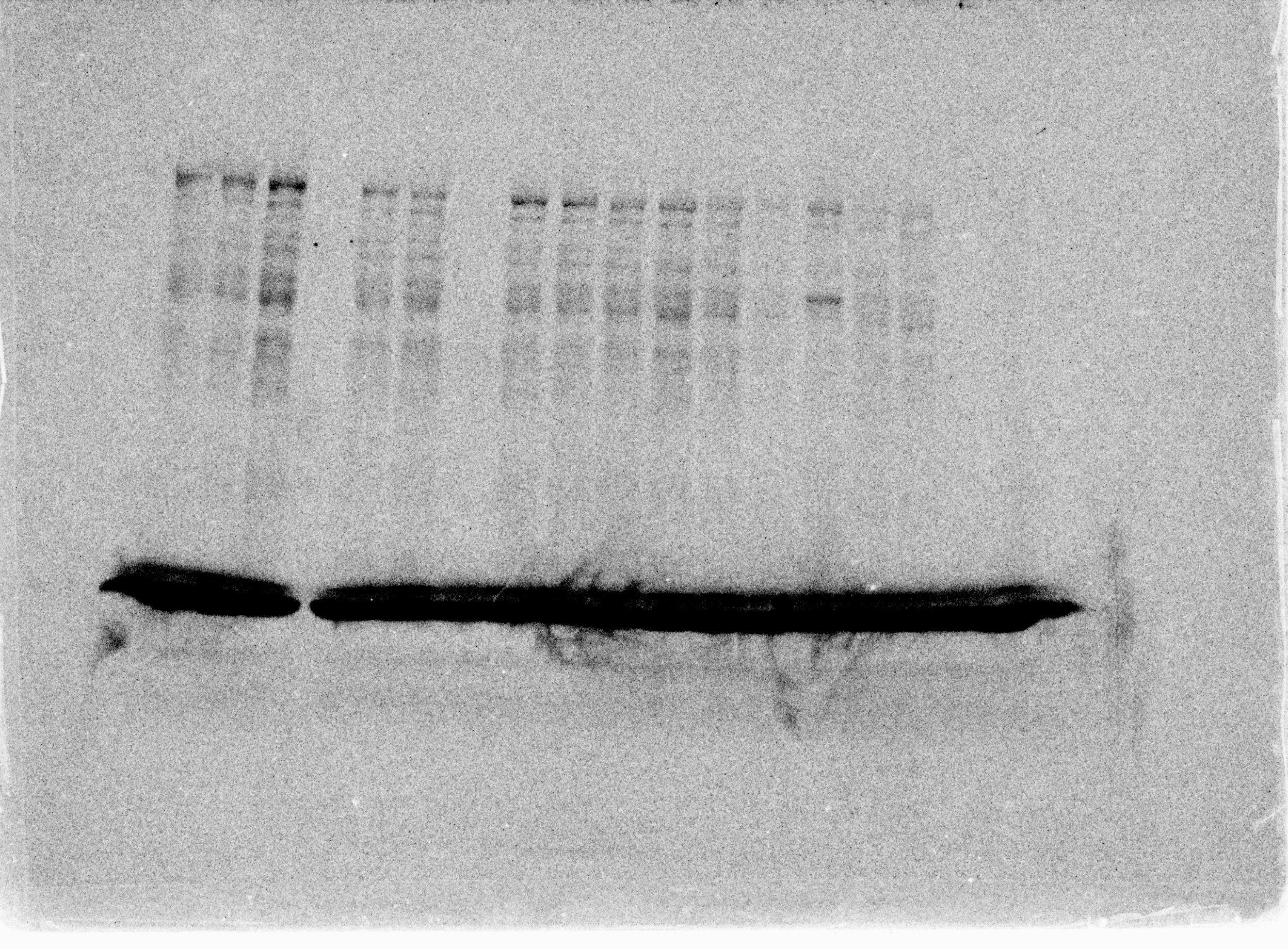

Supplement: Figure 4—figure supplement 2—source data 1. — All the TLCs are marked with the origin (where 20 μL reaction mix is spotted), the acyl-CoA band (closest to origin), the acyl-AMP band (closest to the solvent front), and the free fatty acid band (near the solvent front). All the modified radio-CS-PAGE are marked with the acyl-ACP band and a diffused band, which may be free fatty acids or the acyl-AMP formed in the reaction. (A) Representative TLC images showing that the acyl-AMP formation is minimally or not affected by mutations in the alternate pocket of MxFAAL. (B) A representative modified radio-CS-PAGE image showing the acyl-MxACP formation by alternate pocket mutants of MxFAAL. The multiple bands in the modified radio-CS-PAGE may represent either the degradation products of MxACP-GFP or the multiple unfolded forms of MxACP-GFP in the presence of urea in the gel. (C) Representative TLC images showing the acyl-AMP formation by alternate pocket mutants of RsFAAL. (D) A representative modified radio-CS-PAGE image showing the acyl-RsACP formation by alternate pocket mutants of RsFAAL. (E) Representative TLC images showing the acyl-AMP formation by alternate pocket mutants of MsFAAL32. (F) A representative radio-SDS-PAGE showing the acyl-MsPKS131-1042 formation by the alternate pocket mutants of MsFAAL32. Several mutations of the alternate pocket of different FAALs were generated in this study, which had multiple issues including protein stability, poor or complete loss of biochemical activity, etc., hence were not discussed further, and these mutations are marked by a red asterisk as ‘not part of the study.’. [file elife-70067-fig4-figsupp2-data1.zip › Figure 4—Figure_Supplement_2_source_data1-f.png]

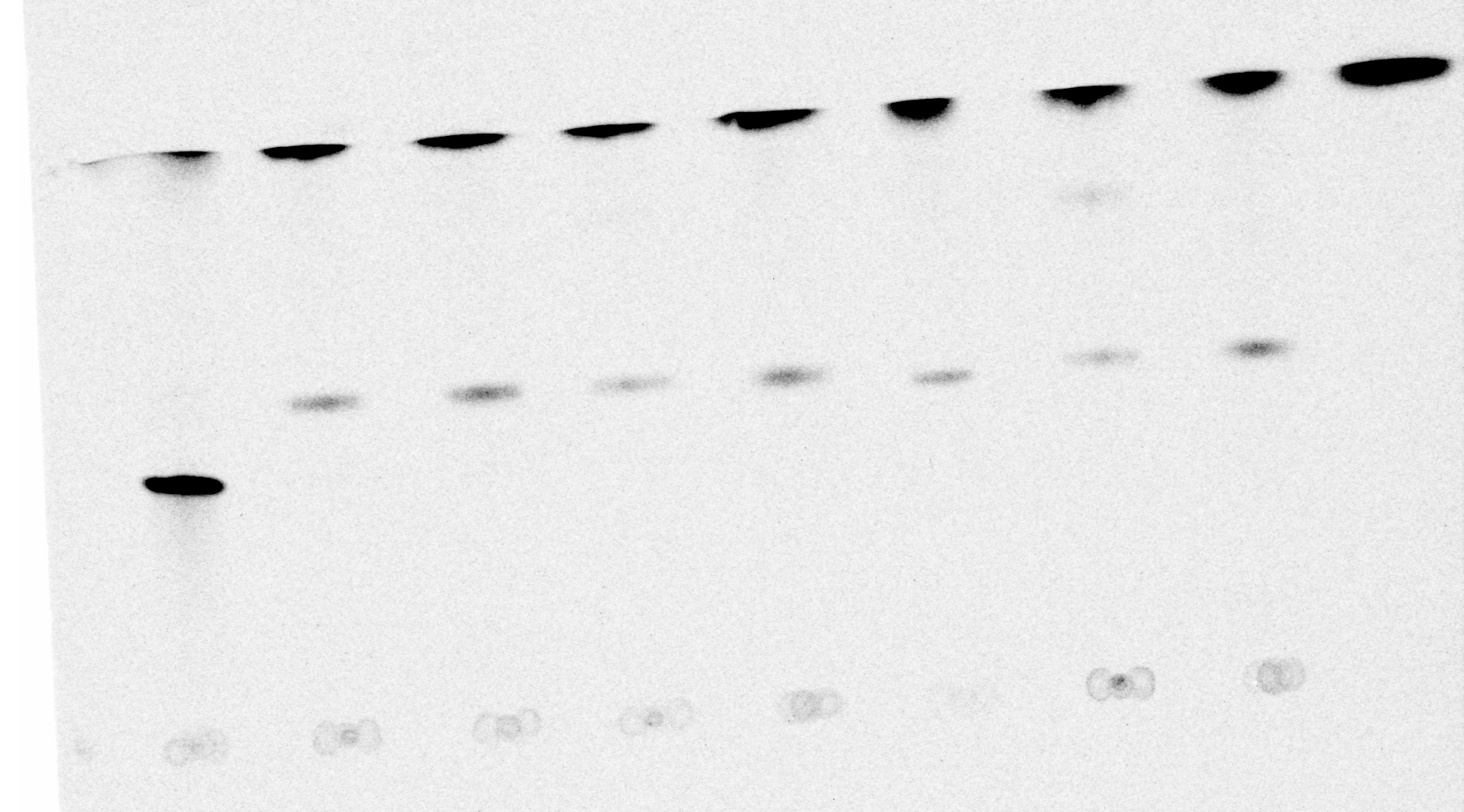

Supplement: Figure 4—figure supplement 2—source data 1. — All the TLCs are marked with the origin (where 20 μL reaction mix is spotted), the acyl-CoA band (closest to origin), the acyl-AMP band (closest to the solvent front), and the free fatty acid band (near the solvent front). All the modified radio-CS-PAGE are marked with the acyl-ACP band and a diffused band, which may be free fatty acids or the acyl-AMP formed in the reaction. (A) Representative TLC images showing that the acyl-AMP formation is minimally or not affected by mutations in the alternate pocket of MxFAAL. (B) A representative modified radio-CS-PAGE image showing the acyl-MxACP formation by alternate pocket mutants of MxFAAL. The multiple bands in the modified radio-CS-PAGE may represent either the degradation products of MxACP-GFP or the multiple unfolded forms of MxACP-GFP in the presence of urea in the gel. (C) Representative TLC images showing the acyl-AMP formation by alternate pocket mutants of RsFAAL. (D) A representative modified radio-CS-PAGE image showing the acyl-RsACP formation by alternate pocket mutants of RsFAAL. (E) Representative TLC images showing the acyl-AMP formation by alternate pocket mutants of MsFAAL32. (F) A representative radio-SDS-PAGE showing the acyl-MsPKS131-1042 formation by the alternate pocket mutants of MsFAAL32. Several mutations of the alternate pocket of different FAALs were generated in this study, which had multiple issues including protein stability, poor or complete loss of biochemical activity, etc., hence were not discussed further, and these mutations are marked by a red asterisk as ‘not part of the study.’. [file elife-70067-fig4-figsupp2-data1.zip › Figure 4—Figure_Supplement_2_source_data1-e2.png]

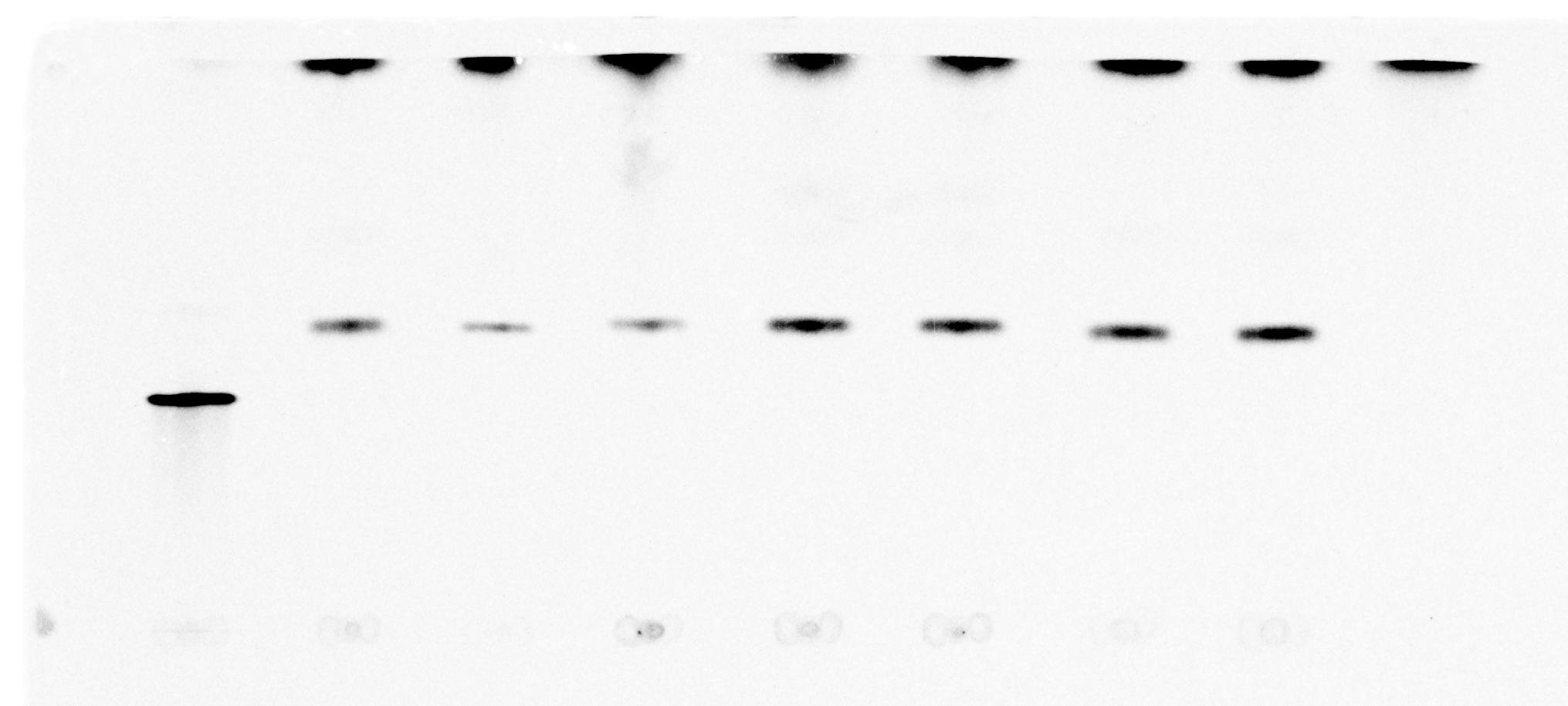

Supplement: Figure 4—figure supplement 2—source data 1. — All the TLCs are marked with the origin (where 20 μL reaction mix is spotted), the acyl-CoA band (closest to origin), the acyl-AMP band (closest to the solvent front), and the free fatty acid band (near the solvent front). All the modified radio-CS-PAGE are marked with the acyl-ACP band and a diffused band, which may be free fatty acids or the acyl-AMP formed in the reaction. (A) Representative TLC images showing that the acyl-AMP formation is minimally or not affected by mutations in the alternate pocket of MxFAAL. (B) A representative modified radio-CS-PAGE image showing the acyl-MxACP formation by alternate pocket mutants of MxFAAL. The multiple bands in the modified radio-CS-PAGE may represent either the degradation products of MxACP-GFP or the multiple unfolded forms of MxACP-GFP in the presence of urea in the gel. (C) Representative TLC images showing the acyl-AMP formation by alternate pocket mutants of RsFAAL. (D) A representative modified radio-CS-PAGE image showing the acyl-RsACP formation by alternate pocket mutants of RsFAAL. (E) Representative TLC images showing the acyl-AMP formation by alternate pocket mutants of MsFAAL32. (F) A representative radio-SDS-PAGE showing the acyl-MsPKS131-1042 formation by the alternate pocket mutants of MsFAAL32. Several mutations of the alternate pocket of different FAALs were generated in this study, which had multiple issues including protein stability, poor or complete loss of biochemical activity, etc., hence were not discussed further, and these mutations are marked by a red asterisk as ‘not part of the study.’. [file elife-70067-fig4-figsupp2-data1.zip › Figure 4—Figure_Supplement_2_source_data1-e1.png]

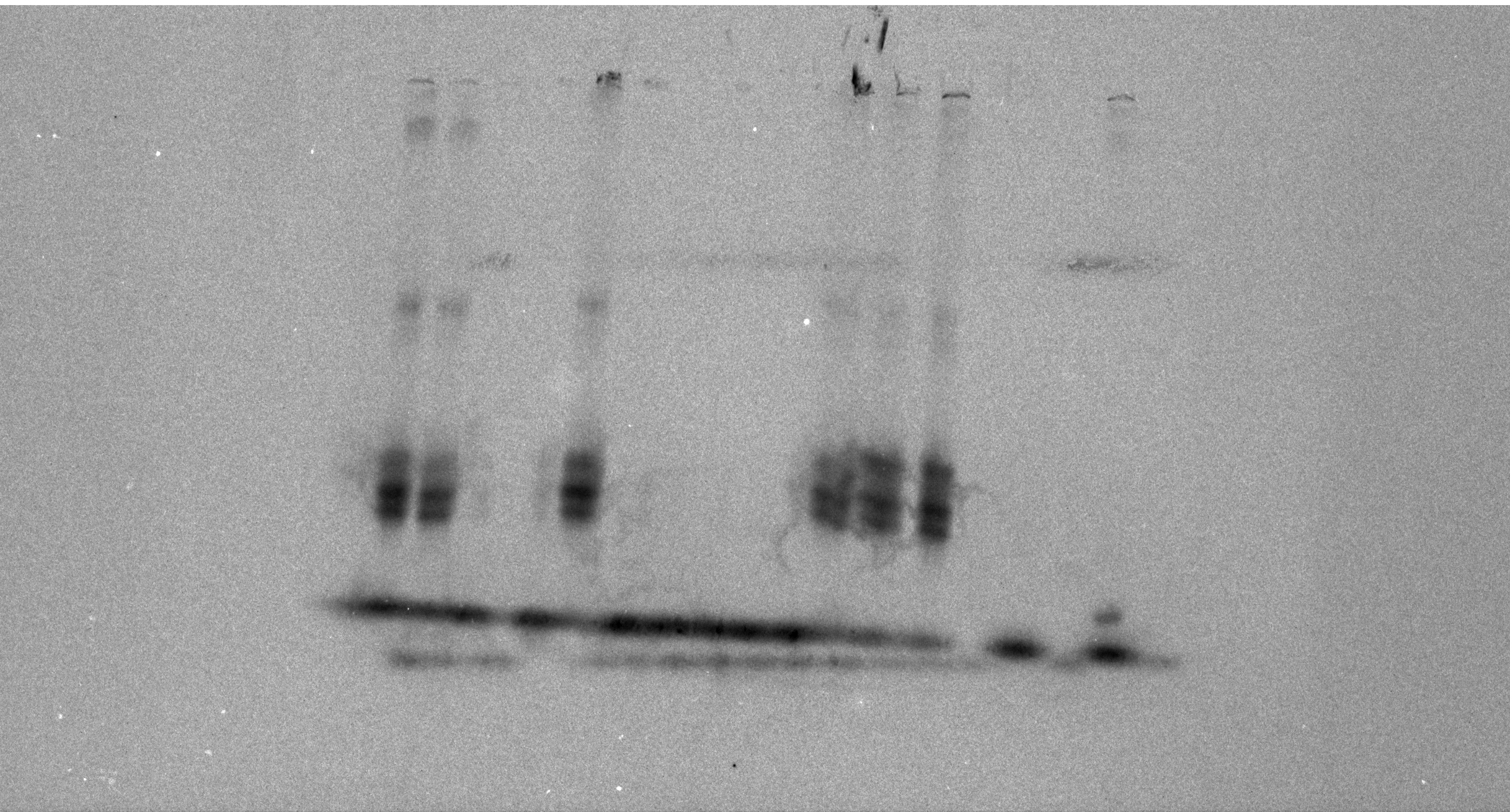

Supplement: Figure 4—figure supplement 2—source data 1. — All the TLCs are marked with the origin (where 20 μL reaction mix is spotted), the acyl-CoA band (closest to origin), the acyl-AMP band (closest to the solvent front), and the free fatty acid band (near the solvent front). All the modified radio-CS-PAGE are marked with the acyl-ACP band and a diffused band, which may be free fatty acids or the acyl-AMP formed in the reaction. (A) Representative TLC images showing that the acyl-AMP formation is minimally or not affected by mutations in the alternate pocket of MxFAAL. (B) A representative modified radio-CS-PAGE image showing the acyl-MxACP formation by alternate pocket mutants of MxFAAL. The multiple bands in the modified radio-CS-PAGE may represent either the degradation products of MxACP-GFP or the multiple unfolded forms of MxACP-GFP in the presence of urea in the gel. (C) Representative TLC images showing the acyl-AMP formation by alternate pocket mutants of RsFAAL. (D) A representative modified radio-CS-PAGE image showing the acyl-RsACP formation by alternate pocket mutants of RsFAAL. (E) Representative TLC images showing the acyl-AMP formation by alternate pocket mutants of MsFAAL32. (F) A representative radio-SDS-PAGE showing the acyl-MsPKS131-1042 formation by the alternate pocket mutants of MsFAAL32. Several mutations of the alternate pocket of different FAALs were generated in this study, which had multiple issues including protein stability, poor or complete loss of biochemical activity, etc., hence were not discussed further, and these mutations are marked by a red asterisk as ‘not part of the study.’. [file elife-70067-fig4-figsupp2-data1.zip › Figure 4—Figure_Supplement_2_source_data1-d.png]

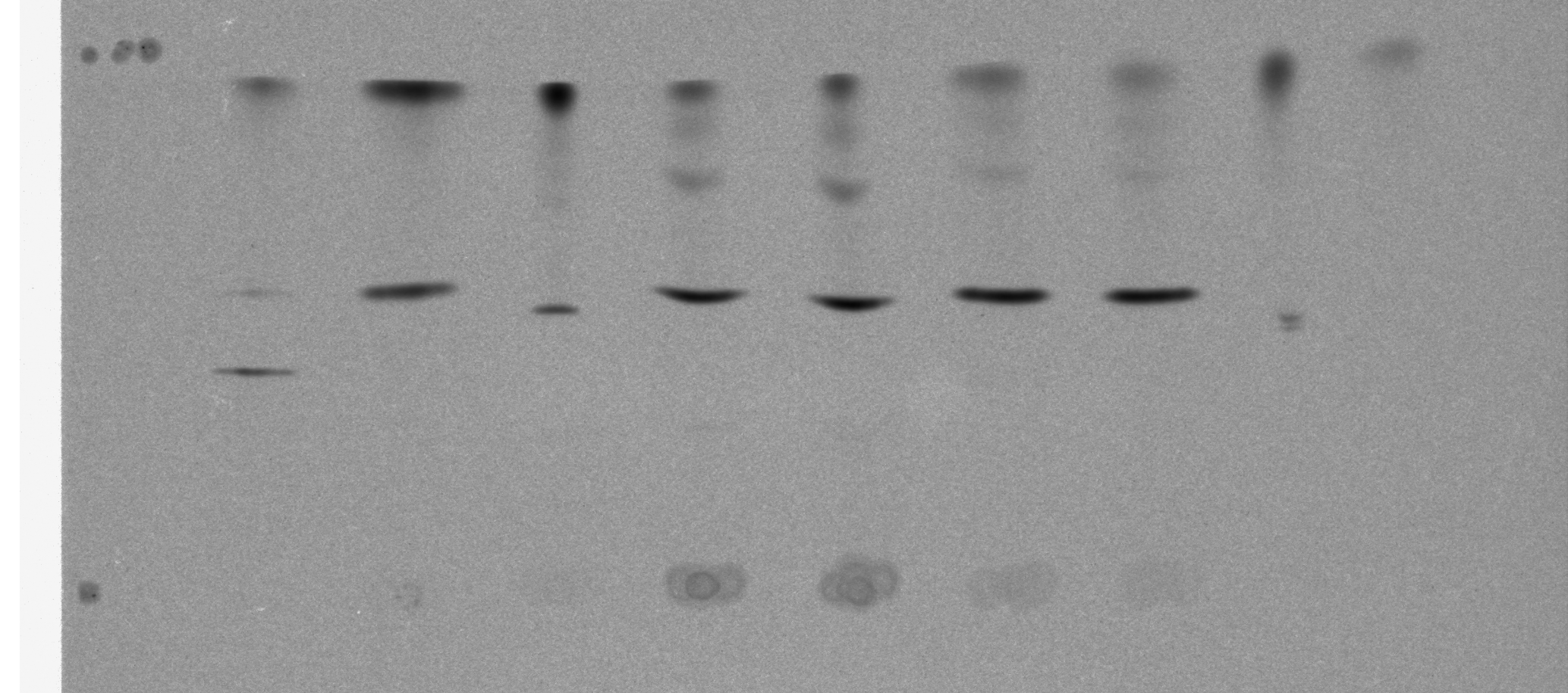

Supplement: Figure 4—figure supplement 2—source data 1. — All the TLCs are marked with the origin (where 20 μL reaction mix is spotted), the acyl-CoA band (closest to origin), the acyl-AMP band (closest to the solvent front), and the free fatty acid band (near the solvent front). All the modified radio-CS-PAGE are marked with the acyl-ACP band and a diffused band, which may be free fatty acids or the acyl-AMP formed in the reaction. (A) Representative TLC images showing that the acyl-AMP formation is minimally or not affected by mutations in the alternate pocket of MxFAAL. (B) A representative modified radio-CS-PAGE image showing the acyl-MxACP formation by alternate pocket mutants of MxFAAL. The multiple bands in the modified radio-CS-PAGE may represent either the degradation products of MxACP-GFP or the multiple unfolded forms of MxACP-GFP in the presence of urea in the gel. (C) Representative TLC images showing the acyl-AMP formation by alternate pocket mutants of RsFAAL. (D) A representative modified radio-CS-PAGE image showing the acyl-RsACP formation by alternate pocket mutants of RsFAAL. (E) Representative TLC images showing the acyl-AMP formation by alternate pocket mutants of MsFAAL32. (F) A representative radio-SDS-PAGE showing the acyl-MsPKS131-1042 formation by the alternate pocket mutants of MsFAAL32. Several mutations of the alternate pocket of different FAALs were generated in this study, which had multiple issues including protein stability, poor or complete loss of biochemical activity, etc., hence were not discussed further, and these mutations are marked by a red asterisk as ‘not part of the study.’. [file elife-70067-fig4-figsupp2-data1.zip › Figure 4—Figure_Supplement_2_source_data1-c2.png]
