# Supplementary material for: A universal pocket in fatty acyl-AMP ligases ensures redirection of fatty acid pool away from coenzyme A-based activation
Source: eLife. 2021 Sep 7;10:e70067. doi: 10.7554/eLife.70067 (PMC8460268; doi:10.7554/eLife.70067)
Supplement: Supplementary file 2. — The number of atoms of the protein at a clashing distance is an indicator of the space available in the canonical pocket. A higher number of atoms in FAALs indicates the limited space available in the pocket, while the lower number indicates that it is more accommodative in the case of FACLs. Crystal structures of known FACLs have at least one atom. [file elife-70067-supp2.docx]

|  | **Number of atoms in FAALs at ≤ 2.5 Å from multiple CoA conformations** | | | | | |
| --- | --- | --- | --- | --- | --- | --- |
|  | 1PG4_CoA | 3EQ6_CoA | 3CW9_CoA | 5BSR_CoA | 3NYQ_CoA | **Average** |
| 3PBK | 9 | 9 | 5 | 13 | 2 | 7.6 |
| 5ICR | 8 | 9 | 10 | 10 | 2 | 7.8 |
| 3E53 | 5 | 5 | 9 | 6 | 2 | 5.4 |
| 3KXW | 7 | 4 | 10 | 8 | 2 | 6.2 |
|  |  |  |  |  |  | **7** |
|  | | | | | |  |
|  | **Number of atoms in FACLs at ≤ 2.5 Å from multiple CoA conformations** | | | | | |
|  | 1PG4_CoA | 3EQ6_CoA | 3CW9_CoA | 5BSR_CoA | 3NYQ_CoA | **Average** |
| 1PG4 | 0 | 0 | 5 | 3 | 0 | 1.6 |
| 3EQ6 | 4 | 0 | 4 | 3 | 0 | 2.2 |
| 3CW9 | 6 | 0 | 0 | 1 | 0 | 1.4 |
| 5BSR | 5 | 1 | 1 | 0 | 0 | 1.4 |
| 3R44 | 3 | 0 | 0 | 0 | 1 | 0.8 |
| 3A9V | 7 | 0 | 0 | 0 | 0 | 1.4 |
| 3NYQ | 8 | 1 | 2 | 3 | 0 | 2.8 |
| 4DG9 | 0 | 0 | 1 | 0 | 0 | 0.2 |
|  | | | | | | **1** |
|  | | | | | |  |
|  | **Number of atoms in FAALs at ≤ 2.0 Å from multiple CoA conformations** | | | | | |
|  | 1PG4_CoA | 3EQ6_CoA | 3CW9_CoA | 5BSR_CoA | 3NYQ_CoA | **Average** |
| 3PBK | 1 | 7 | 2 | 7 | 2 | 3.8 |
| 5ICR | 4 | 3 | 5 | 6 | 1 | 3.8 |
| 3E+53 | 2 | 4 | 6 | 4 | 2 | 3.6 |
| 3KXW | 3 | 1 | 6 | 6 | 1 | 3.4 |
|  | | | | | | **4** |
|  | | | | | |  |
|  | **Number of atoms in FACLs at ≤ 2.0 Å from multiple CoA conformations** | | | | | |
|  | 1PG4_CoA | 3EQ6_CoA | 3CW9_CoA | 5BSR_CoA | 3NYQ_CoA | **Average** |
| 1PG4 | 0 | 0 | 3 | 2 | 0 | 1 |
| 3EQ6 | 3 | 0 | 3 | 2 | 0 | 1 |
| 3CW9 | 5 | 0 | 0 | 0 | 0 | 1 |
| 5BSR | 4 | 0 | 1 | 0 | 0 | 1 |
| 3R44 | 1 | 0 | 0 | 0 | 0 | 0 |
| 3A9V | 4 | 0 | 0 | 0 | 0 | 1 |
| 3NYQ | 4 | 0 | 0 | 1 | 0 | 1 |
| 4DG9 | 3 | 0 | 1 | 0 | 0 | 0 |
|  | | | | | | **1** |
