# Supplementary material for: A universal pocket in fatty acyl-AMP ligases ensures redirection of fatty acid pool away from coenzyme A-based activation
Source: eLife. 2021 Sep 7;10:e70067. doi: 10.7554/eLife.70067 (PMC8460268; doi:10.7554/eLife.70067)
Supplement: Supplementary file 3. — The phyla of the eubacteria where these clusters are found, the name of the metabolites, the GenBank ID of the biosynthesis cluster, the FAAL (stand-alone or fused) along with their GenBank ID (common name included) and the references where they have been described are detailed. [file elife-70067-supp3.docx]

| **Phylum** | **Bioactive metabolite** | **Biosynthetic cluster GenBank ID** | **FAAL (GenBank ID)** | **Reference** |
| --- | --- | --- | --- | --- |
| **Proteobacteria** | Phenylnannolone | KF739396.1 | Phn2 (AHN85651.1) | (Bouhired et al., 2014) |
|  | Ambruticin | DQ897667.1 | AmbG (ABK32262.1) | (Hemmerling et al., 2018; Julien et al., 2006) |
|  | Alklyresorcylic acids | NC_008095.1 | FtpD (WP011556561.1) | (Hayashi et al., 2011) |
|  | Caryoynencin | CP022213.1 (3094977 to 3108181) | CayA (AWY55460.1) | (Ross et al., 2014) |
|  | Vioprolide | MH108942.1 | VioA (AWI62626.1) | (Auerbach et al., 2018) |
|  | Tambjamine YP1 | AAOH01000002.1 (146524 to 172121) | TamA (EAR29369.1) | (Marchetti et al., 2018) |
|  | Ralsolamycin | AL646053.1 (777576 to 820777) | RSp0641 (CAD17792.1) | (Spraker et al., 2016) |
|  | Micacocidin | AL646052.1 (1941939 to 1979076) | RSc1806 (CAD15508.1) | (Kage et al., 2013; Kreutzer et al., 2011) |
| **Cyanobacteria** | Puwainaphycin | KM078884.1 | PuwC (AIW82280.1) | (Mares et al., 2014) |
|  | Olefin | CP000951.1 | SYNPCC7002_A1173 (ACA99172.1) | (Zhu et al., 2018) |
|  | Jamaicamide | AY522504.1 | JamA (AAS98774.1) | (Edwards et al., 2004) |
|  | Hectochlorin | AY974560.1 | HctA (AAY42393.1) | (Kleigrewe et al., 2015) |
|  | Microginin | NZ_HE973145.1 (58948 to 82880) | MICAD_RS03045 (WP_002774031.1) | (Humbert et al., 2013) |
| **Actinobacteria** | Mycolic acid | NC_000962.3 (4255945 to 4263066) | MtFAAL32 (NP_218318.1) | (Gavalda et al., 2009; Leger et al., 2009) |
|  | PDIM | NC_000962.3 (3276380 to 3302455) | MtFAAL29 (NP_217466.3)  MtFAAL22 (NP_217464.1)  MtFAAL28 (NP_217457.1) | (Simeone et al., 2010; Trivedi et al., 2005) |
|  | Glycopeptidolipid | AY439015.3 | MsFAAL28 (ABC70854.1) | (Vats et al., 2012) |
|  | Daptomycin | AY787762.1 | DptE (AAX31555.1) | (Baltz et al., 2021; Wittmann et al., 2008) |
|  | Taromycin A | KF301601.1 | - (AHH53502.1) | (Yamanaka et al., 2014) |
| **Firmicutes** | Mycosubtilin | AF184956.1 | MycA (AAF08795.1) | (Duitman et al., 1999) |
|  | Bacillomycin | CP000560.1 (1867976 to 1914123) | BmyA (ABS74181.1) | (Koumoutsi et al., 2004; Lu et al., 2019) |
