# Supplementary material for: A universal pocket in fatty acyl-AMP ligases ensures redirection of fatty acid pool away from coenzyme A-based activation
Source: eLife. 2021 Sep 7;10:e70067. doi: 10.7554/eLife.70067 (PMC8460268; doi:10.7554/eLife.70067)
Supplement: Supplementary file 4. [file elife-70067-supp4.docx]

| **Lineage**  **(Schoch et al., 2020)** | **Organisms** | **Type of FAAL-like domain (domain organization)** |
| --- | --- | --- |
| **Amoebozoa** | L8HIJ4\|*Acanthamoeba castellanii* | Bacterial/Plant FAAL |
| **Ancyromonadida** | **-** | **-** |
| **Apusozoa** | **-** | **-** |
| **Breviatea** | **-** | **-** |
| **CRuMs** | **-** | **-** |
| **Cryptophyceae** | **-** | **-** |
| **Discoba** | **-** | **-** |
| **Glaucocystophyceae** | **-** | **-** |
| **Haptista** | R1DLD1\|*Emiliania huxleyi* | Bacterial/Plant FAAL |
| **Hemimastigophora** | **-** | **-** |
| **Malawimonadida** | **-** | **-** |
| **Metamonada** | **-** | **-** |
| **Opisthokonta** | | |
| - Aphelida | **-** | **-** |
| - Choanoflagellata | A9UP98\|*Monosiga brevicollis*;  F2U8X9\|*Salpingoeca rosetta* | Opisthokonta FAAL-like domains  (two-domain) |
| - Filasterea | A0A0D2UCG9\|*Capsaspora owczarzaki* | Opisthokonta FAAL-like domains  (three-domain) |
| - Fungi | Many organisms (except Basidiomycetes) | Opisthokonta FAAL-like domains  (three-domain) |
| - Ichthyosporea | **-** | **-** |
| - Metazoa |  | Opisthokonta FAAL-like domains  (three-domain) |
| - Rotosphaerida | **-** | **-** |
| Rhodelphea | **-** | **-** |
| Rhodophyta | **-** | **-** |
| **SAR group** |  |  |
| - Stramenophiles - Alveolata - Rhizaria | A0A067C5L3\|*Saprolegnia parasitica*;  A0A1V9Z9Z4\|*Achlya hypogyna* | Bacterial/Plant FAAL |
|  | V4ZF64\|Toxoplasma gondii;  U6GWK7\|*Eimeria acervuline* | Bacterial/Plant FAAL |
|  | **-** | **-** |
| **Viridiplantae** | A0A445HLB4\|*Glycine soja*; A0A3Q0EXH4\|*Vigna radiata* Q01KB0 \|*Oryza sativa*; UPI000CE285CA\|*Quercus suber*; UPI0011E53342\|*Rhodamnia argentea* | Bacterial/Plant Bacterial/Plant fused to HemY/Catalase  Di-domain form (tandem FAAL-like) |
