# Supplementary material for: A universal pocket in fatty acyl-AMP ligases ensures redirection of fatty acid pool away from coenzyme A-based activation
Source: eLife. 2021 Sep 7;10:e70067. doi: 10.7554/eLife.70067 (PMC8460268; doi:10.7554/eLife.70067)
Supplement: Supplementary file 5. — (A) Residues from representative fatty acyl/aryl-CoA ligases (FACLs) (HsFACL, 3EQ6; SeACS, 1PG4; MtFACL13, 3R44; AfFACL, 3G7S; EcFACL, homology model from AlphaFold Protein Structure Database) and fatty acyl-AMP ligases (FAALs) (MtFAAL28, 3E53; MsFAAL32, 5ICR; EcFAAL, 3PBK; RsFAAL, homology model generated using MODELLER) at 4.5 Å distance from coenzyme A (CoA) bound to SeACS (PDB: 1PG4) and HsFACL (PDB: 3EQ6) are identified by structural superposition and tabulated. The FAALs and FACLs included here are those that were used for biochemical analysis along with the availability of their crystal structures. The stick representation of CoA (yellow) is shown along the table with entry and active site marked. The variability of residues along the CoA-binding site results in differential orientation of 4'-phosphopantetheine arm during catalysis. For instance, compare the biochemical profiles of F284A/M233A mutation in MsFAAL32 and F265A/M217A in RsFAAL with respect to residues in the vicinity of the residues chosen for mutation. The conserved phenylalanine in FAALs (F284 of MsFAAL32 and F265 in RsFAAL) is flanked by different residues (H288 in MsFAAL32 and A269 in RsFAAL). Similarly, the conserved methionine of FAALs (M233 of MsFAAL32 and M217 of RsFAAL) is abutted by different residues (S314 in MsFAAL32 and A294 in RsFAAL). (B) A tabulation summarizing the biochemical profiles of various mutations generated in the study. Residue numbers for EcFAAL re used as reference in tables for the ‘gain of function in FAALs through mutations in canonical pocket’ and ‘loss of function in FAALs through mutations in alternate pocket,’ while MtFACL13 is used as reference in tables for the ‘loss of function in FAALs through mutations in canonical pocket.’ A tick (✓) mark indicates that the biochemical activity has been performed in accordance with the hypothesis, which is mentioned in the title of each of the table. A hyphen (-) sign indicates that the mutation did not work as per the hy [file elife-70067-supp5.docx]

| **FACLs** | | | | |  |  | **FAALs** | | | |  |  | **Entry**  **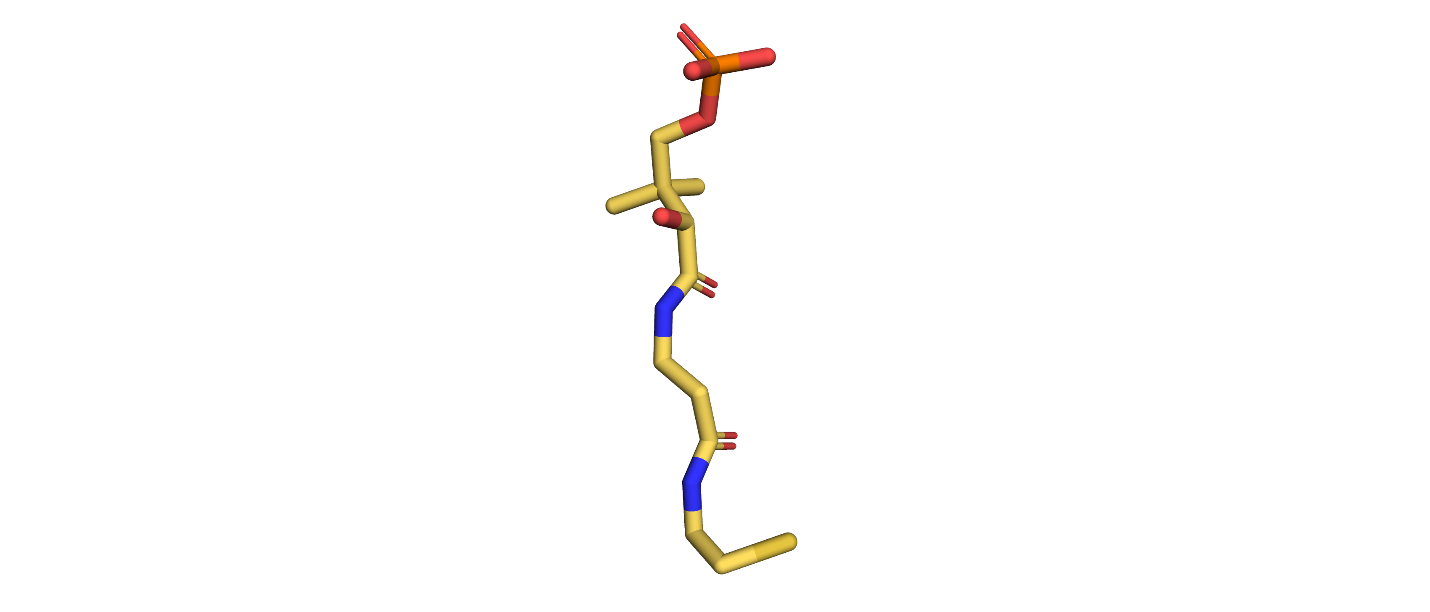**  **Active site** |
| --- | --- | --- | --- | --- | --- | --- | --- | --- | --- | --- | --- | --- | --- |
| **StACS (1PG4)** | **HsFACL**  **(3EQ6)** | **AfFACL (3G7S)** | **MtFACL13 (3R44)** | **EcFACL (model)** |  |  | **MtFAAL28**  **(3E53)** | **EcFAAL**  **(3PBK)** | **MsFAAL32**  **(5ICR)** | **RsFAAL**  **(model)** |  |  |  |
| R191 | - | - | - | - |  |  | - | - | - | - |  |  |  |
| G332 | K290 | M252 | Q230 | N284 |  |  | P244 | T252 | P253 | P238 |  |  |  |
| P334 | D292 | N254 | D232 | D287 |  |  | F247 | F255 | F256 | F241 |  |  |  |
| V333 | F291 | F253 | F231 | R286 |  |  | L248 | A256 | V257 | I242 |  |  |  |
| A363 | M317 | V279 | F257 | A312 |  |  | L275 | L283 | H288 | A269 |  |  |  |
| A360 | V314 | A276 | I254 | L309 |  |  | A272 | G280 | A285 | G266 |  |  |  |
| T359 | I313 | P275 | A253 | T308 |  |  | F271 | F279 | F284 | F265 |  |  |  |
| A357 | A311 | V273 | V251 | V306 |  |  | P269 | P277 | P282 | P263 |  |  |  |
| Y355 | M309 | W271 | G249 | T304 |  |  | A267 | V275 | V280 | G261 |  |  |  |
| V386 | V337 | G302 | G278 | G333 |  |  | G299 | G307 | G313 | G293 |  |  |  |
| G387 | G338 | A303 | G279 | G334 |  |  | S300 | A308 | S314 | A294 |  |  |  |
| T311 | L267 | A232 | A210 | F263 |  |  | M223 | M231 | M233 | M217 |  |  |  |
| V310 | I266 | S231 | V209 | I262 |  |  | D222 | D230 | D232 | D216 |  |  |  |
| W309 | W265 | H230 | H208 | H261 |  |  | H221 | H229 | H231 | H215 |  |  |  |
| D306 | D262 | P227 | P205 | P258 |  |  | P218 | P226 | P228 | P212 |  |  |  |
| A305 | S261 | M226 | L204 | L257 |  |  | L217 | L225 | L227 | L211 |  |  |  |
| T304 | I260 | C225 | P203 | A256 |  |  | W216 | W224 | W226 | W210 |  |  |  |

(**a**)

(**b**)

| **Gain of function in FAALs through mutations in canonical pocket** | | | |
| --- | --- | --- | --- |
| **Residues mutated** | ***Ec*FAAL** | ***Ms*FAAL32** | ***Rs*FAAL** |
| F279A | 🗸 | - | 🗸 |
| M231A | 🗸 | - | 🗸 |
| ∆FSH_254-257_ | 🗸 | 🗸 | 🗸 |
| F279A/M231A | 🗸 | - | 🗸 |
| F279A/M231A/A256G | 🗸 | 🗸 | 🗸 |

| **Loss of function in FACLs through mutations in canonical pocket** | | | |
| --- | --- | --- | --- |
| **Residues mutated** | ***Mt*FACL13** | ***Af*FACL** | ***Ec*FACL** |
| A253F | 🗸 | - | - |
| SQFL_ins_ | 🗸 | - | 🗸 |
| A253F/ SQFL_ins_ | 🗸 | - | - |
| A253F/ A210M | - | 🗸 | - |
| A253F/ A210M/ SQFL_ins_ | 🗸 | 🗸 | 🗸 |

| **Loss of function in FAALs through mutations in alternate pocket** | | | |
| --- | --- | --- | --- |
| **Residues mutated** | ***Ec*FAAL** | ***Rs*FAAL32** | ***Mx*FAAL** |
| T83F | 🗸 | 🗸 | - |
| T83R | 🗸 | NA | 🗸 |
| P107F | 🗸 | 🗸 | 🗸 |
| P107R | 🗸 | 🗸 | 🗸 |
| T252F | 🗸 | 🗸 | - |
| T252R | 🗸 | - | 🗸 |
